# Cannibalism shapes biofilm structure and composition in *Bacillus subtilis*

**DOI:** 10.1128/mbio.00525-26

**Published:** 2026-06-15

**Authors:** Lena Friebel, Jan-Philipp Knepper, Nathalie Sofie Becker, Gorkhmaz Abbaszade, Kathrin Stückrath, Jens Soltwisch, Susann Müller, Klaus Dreisewerd, Thorsten Mascher

**Affiliations:** 1 TUD University of Technology Dresden, Chair of General Microbiology 9169, Dresden, Germany; 2 Institute of Hygiene, University of Münster 9185, Münster, Germany; 3 Interfaculty Institute of Microbiology and Infection Medicine, University of Tübingen 9188, Tübingen, Germany; 4 Department of Applied Microbial Ecology, Helmholtz Centre for Environmental Research-UFZ 542364 , Leipzig, Germany; University of Ljubljana, Biotechnical Faculty, Ljubljana, Slovenia

**Keywords:** programmed cell death, antimicrobial peptides, multicellularity, differentiation, *Bacillus subtilis*, sporulation, MALDI-mass spectrometry imaging, flow cytometry

## Abstract

In *Bacillus subtilis* colony biofilms, phenotypic diversification confers tissue-like properties and enhanced competitive fitness within a structural framework that allows both colony expansion and long-term survival via endospore formation. Cannibalism is a sporulation delay strategy, in which one subpopulation produces the sporulation delay protein SDP, the sporulation killing factor SKF, and the epipeptide EPE. These toxins are thought to lyse susceptible nonproducers, thereby releasing nutrients to prevent premature sporulation. However, the molecular mechanisms orchestrating this bacterial type of programmed cell death during biofilm development are poorly understood. Here, we comprehensively characterized mutants defective in either toxin production or the corresponding autoimmunity by a multiscale approach, combining luminescence reporters, colony biopsy, multi-parameter flow cytometry, and MALDI-mass spectrometry imaging to resolve cannibalism function and distribution. The toxins are produced in distinct, only partially overlapping areas of the colony, and are interdependent in their spatial distribution. Both EPE and SDP, but not SKF, are crucial for delaying sporulation. Loss of EPE or SDP autoimmunity resulted in severe morphological changes and stress-induced occurrence of suppressor mutants. The absence of all three toxins led to small, hyper-sporulating colonies with excessive wrinkle formation, indicating that cannibalism is essential for maintaining biofilm structure and lateral expansion. Our results provide the first evidence for the complex interactions between the three cannibalism toxins that shape biofilm architecture through bacterial programmed cell death. Localized toxin production and its spatial distribution affect the spatiotemporal organization, morphology, and subpopulation dynamics within *B. subtilis* biofilms.

**IMPORTANCE:**

Programmed cell death (PCD) is a ubiquitous and crucial mechanism to structure eukaryotic multicellular tissues. PCD-like processes have also been described in bacteria, but their contribution to multicellular development is poorly understood. Cannibalism in *Bacillus subtilis* has been described as a sporulation delay strategy, in which one subpopulation produces antimicrobial peptides that kill susceptible nonproducing siblings. Their lysis is thought to release nutrients that delay the sporulation in the producing subpopulation. This study comprehensively analyses the role of the three cannibalism toxins in shaping colony biofilms. By combining MALDI-mass spectrometry imaging, colony biopsy, flow cytometry, and luminescence reporters, we demonstrate that cannibalism toxins are crucial for biofilm structure. They show a discrete and interdependent localization within the colonies. While cannibalism inhibits sporulation and causes severe envelope stress within biofilms, our data challenge the established role of cannibalism-dependent killing as the mechanism behind this sporulation delay.

## INTRODUCTION

Biofilms as a collective mode of bacterial life appeared over three billion years ago on our planet (1) and likely evolved as a survival strategy in response to the harsh environmental conditions of early Earth. This evolutionary step allowed bacterial cells to thrive and survive in a self-produced, matrix-protected, and surface-adherent microenvironment, in which collective behaviors and differentiated multicellular traits could develop (2).

The soil-dwelling, gram-positive bacterium *Bacillus subtilis* is a model organism for studying (multi)cellular differentiation and development in complex biofilms (3). A trademark is its ability to transition from a motile to a sessile lifestyle, in which it forms dormant endospores within the context of differentiated biofilms (4). *B. subtilis* can form different types of biofilms depending on the respective environment: pellicle biofilms at the liquid-air interface, submerged surface-attached biofilms, or colony biofilms at the solid-air interface (5). Within *B. subtilis* biofilms, genetically identical cells differentiate into distinct cell types in a spatiotemporally controlled manner—a response to specific microenvironments, e.g., based on the availability of nutrients, which is guided by tightly regulated gene expression programs and influenced by stochastic gene expression and genetic noise (6–12). Central to these processes is the master regulator of sporulation, Spo0A, which orchestrates cell differentiation and ultimately endospore formation upon nutrient depletion (13–16). Spo0A becomes activated through a multicomponent phosphorelay (17), resulting in increasing levels of phosphorylated Spo0A (Spo0A~P) over time (18, 19). As a consequence, it gradually silences regulatory repression of its counterplayer, the transition state regulator AbrB, which suppresses premature activation of stationary phase traits (20). Low levels of Spo0A~P trigger the differentiation of cells into producing the extracellular matrix (ECM), essential for biofilm formation and protecting the cells within (21–23). This subpopulation then displays elevated levels of matrix-associated gene expression in a SinR-dependent manner (24), resulting in the production of ECM, which comprises exopolysaccharides, amyloid TasA fibers, and the hydrophobin-like protein BslA (5, 25). Interestingly, matrix-producing cells have also been reported to exhibit characteristics of cannibalistic cells (26).

Cannibalism is a social behavior in *B. subtilis* that emerges during the post-exponential growth phase, that is, at low Spo0A~P levels (27). It is thought to delay sporulation by enabling a subpopulation of cells to produce cannibalism toxins that lyse neighboring non-cannibalistic cells. The released nutrients are then consumed by the producers to resume growth (28–30). This process is mediated by three ribosomally synthesized and post-translationally modified antimicrobial peptides (RiPPs): the sporulation killing factor SKF, the sporulation delaying protein SDP, and the more recently described epipeptide EPE (13, 30–32).

The *skfABCEFGH* operon contains the structural gene for the SKF toxin (*skfA*), genes for the toxin maturation machinery (*skfB*, *skfC*), an ATP-binding cassette (ABC) transporter that mediates autoimmunity to SKF (*skfEF*), as well as two genes of yet unknown function (*skfG*, *skfH*) (30, 33). The *sdpABC-sdpIR* locus consists of the toxin-encoding gene *sdpC*; the gene products of *sdpA* and *sdpB* are required for toxin maturation, while *sdpIR* encodes the autoimmunity, with SdpR being a transcriptional repressor and SdpI an SDP-sequestering lipoprotein (29). Within the *epeXEPAB* operon, *epeX* encodes the prepropeptide for a secreted antimicrobial peptide (EPE), *epeE* encodes a radical S-adenosyl-L-methionine (SAM) epimerase, which is necessary for maturation and processing of the EPE toxin, together with the transmembrane peptidase, encoded by *epeP*. The genes *epeAB* encode an ABC transporter that mediates autoimmunity against intrinsically produced EPE (32, 34). While the exact mode of action of SKF remains unclear, SDP collapses the proton motive force and triggers autolysis (35), whereas EPE dissipates membrane potential, reduces membrane fluidity, and induces lipid domain formation (32). However, all three toxins exhibit roles beyond their canonical function in sporulation delay: SKF and SDP are implicated in interspecies competition, for example, by strongly inhibiting foreign *Bacillus* species, such as *B. simplex*, while EPE impacts intra-species interactions and kin selection (36–38). As a consequence of producing these destructive molecules, *B. subtilis* mounts a defense strategy known as the cell envelope stress response (CESR) (39, 40). EPE primarily activates the two-component system LiaRS, which upregulates the *liaIH* operon to confer resistance against extrinsically applied EPE (34). BceRS and PsdRS respond to SDP and primarily SKF (33). Both two-component systems regulate ABC transporters, BceAB and PsdAB, respectively, which provide resistance against antimicrobial peptides (41).

In colony biofilms, morphogenesis is tightly linked to mechanical forces generated by growth, matrix production, and localized cell death (42–45). Wrinkle formation and vertical buckling have been associated with programmed cell death (PCD), nutrient limitation, and sporulation. Moreover, these processes depend on the extracellular matrix and surface adhesion (43, 46–50). While the biophysical processes of biofilm formation have been addressed in recent years, little is known about the relationship between structural organization and differentiation processes, such as cannibalism. Technical limitations in resolving chemical signals, cell types, and spatial structure simultaneously within bacterial multicellular aggregates are a major challenge (50), since this requires resolving macroscopic structures, such as colony biofilms, at (or near to) single-cell resolution. However, recent advances in instrumentation and imaging technology now enable multiscale approaches to study biofilm organization at high resolution (51), as demonstrated by this study.

Here, we investigate how cannibalism contributes to the spatial and temporal organization of *B. subtilis* colony biofilms. We provide the first comprehensive, spatially resolved chemical mapping of all three cannibalism toxins within intact colony biofilms, using MALDI-MS imaging similar to that in reference (52. This study establishes a direct link between toxin spatial distribution, colony architecture, and cell-type composition. Under the conditions tested, EPE and SDP, but not SKF, are the dominant sporulation delaying toxins, which refines the classical cannibalism model. Our integrated approach allowed uncovering how cannibalism-induced PCD contributes to biofilm architecture and the resulting emerging multicellular function.

## RESULTS

### Cannibalism affects the morphology of *B. subtilis* colony biofilms

**Fig 1:**
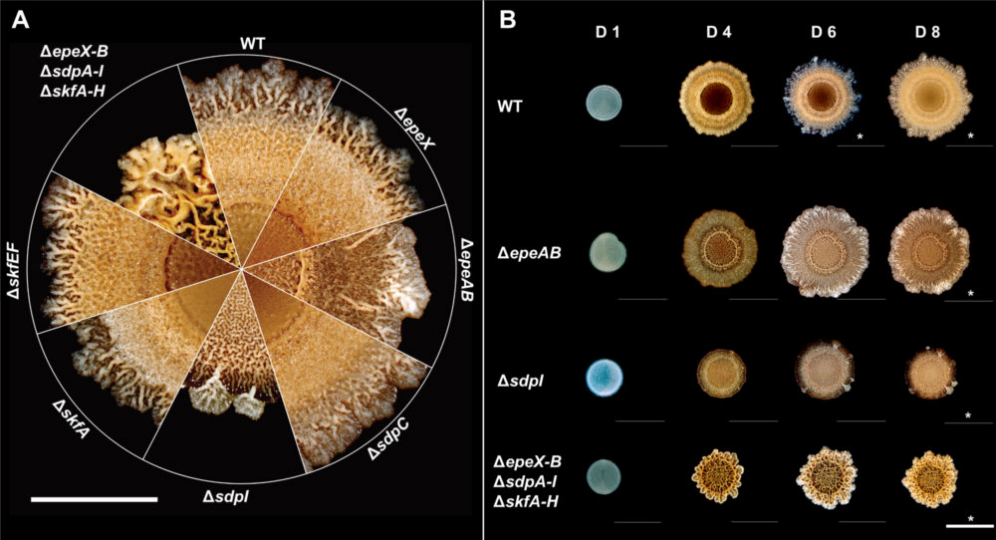
Colony morphologies of *B. subtilis* cannibalistic mutant strains in comparison to the WT. (**A**) Comparative display of 8-day-old biofilms grown on MSgg agar. Clockwise: WT, Δ*epeX*, Δ*epeAB*, Δ*sdpC*, Δ*sdpI*, Δ*skfA*, Δ*skfEF*, and ΔΔΔ (Δ*epeXEPAB* Δ*sdpABC-sdpIR* Δ*skfABCEFGH*). Scale bar indicates 0.5 cm. (**B**) Colony growth and morphology of cannibalistic mutants with distinct phenotypes (Δ*epeAB*, Δ*sdpI*, and ΔΔΔ) in comparison to the WT over the course of 8 days (day 1 [D 1], day 4 [D 4], day 6 [D 6], and day 8 [D 8]). Asterisks mark images captured with 0.8× zoom. Scale bars indicate 1 cm.

Cannibalism is a PCD-type differentiation strategy that involves sacrificing one subpopulation of cells for the benefit of another and therefore operates in the context of multicellular populations. Previous work has indicated that cannibalism is associated with matrix production and hence biofilm formation (26); however, its role in shaping differentiated bacterial tissue has not been examined. We therefore investigated whether cannibalism mutants of *Bacillus subtilis* DK1042, a genetically competent derivative of NCIB3610, exhibited altered morphologies in colony architecture compared to the otherwise isogenic wild-type strain DK1042 (hereafter referred to as WT). Colony morphology was examined by cultivating strains of interest on agar plates of the biofilm-promoting minimal medium MSgg (minimal salts glycerol glutamate) at 28°C and monitoring biofilm formation over 8 days, by capturing daily images (Fig. 1). Cannibalistic mutants were either defective in toxin production due to deletions of the structural genes that encode the pre-pro-peptide (*epeX*, *sdpC*, and *skfA*), or lacked the autoimmunity functions to the respective toxins (*epeAB*, *sdpI*, and *skfEF*). Additionally, we investigated a strain lacking all three cannibalistic toxin-encoding loci (Δ*epeXEPAB* Δ*sdpABCRI* Δ*skfABCEFGH*, abbreviated as ΔΔΔ, and referred to as the hypo-cannibalistic mutant).

Colonies of the WT formed circular biofilms with an undulating edge, characterized by vein-like, intertwined, brownish three-dimensional (3D) structures, in agreement with previous descriptions (4). Three distinct zones were apparent: (i) the center, marking the inoculation site, separated by a ring-like structure from (ii) the inner zone, which was beige in color, primarily flat, but slightly elevated toward (iii) the outer zone. The outer zone was highly structured, featuring pronounced wrinkles extending to the colony’s edge, with a more brownish-gray hue compared to the other zones.

Deletion of individual toxin-encoding genes (*epeX*, *sdpC*, or *skfA*) resulted in only minor morphological changes, like a rather reddish tint across colonies of the *epeX* mutant (Fig. 1A). Similar minor effects on morphology had been observed for an *epeXEPAB* mutant strain (38). The *sdpC* mutant exhibited reduced wrinkle formation in the outer zone, resulting in a flatter, broader surface. Morphological differences in other mutants became apparent from day 4 onward (Fig. 1B). Deletion of the autoimmunity genes for EPE (Δ*epeAB*) or SDP (Δ*sdpI*) caused pronounced alterations in colony size, pigmentation, and structure. In contrast, Δ*skfEF* exhibited no major effects and retained a WT-like colony appearance (Fig. 1A; Fig. S1). The *sdpI* mutant particularly displayed highly convoluted, brain-like folds and poorly defined zonation. The *epeAB* mutant also showed a color shift toward brown across the entire colony, although all three zones remained distinguishable. Both Δ*epeAB* and Δ*sdpI* biofilms exhibited sporadic white outgrowths in the outer zone (Fig. 1; Fig. S1 and S4). The most striking phenotype was observed in the hypo-cannibalistic mutant (ΔΔΔ). Its colonies exhibited excessive wrinkling, increased vertical growth, and markedly reduced lateral expansion (Fig. 1; Fig. S1). Wrinkles were broader, more irregular, and extended across nearly the entire colony surface, in contrast to the more localized wrinkling observed in WT biofilms. Taken together, these observations indicate that while the loss of individual cannibalism toxins has a limited impact on colony morphology, disruption of toxin autoimmunity or complete loss of cannibalism leads to severe defects in colony architecture, size, and surface patterning. These results establish cannibalism as a key determinant of colony morphology and provide a framework for linking structural phenotypes to toxin distribution and cellular differentiation in subsequent analyses.

### Cannibalism toxins show unique and interdependent distribution patterns

**Fig 2:**
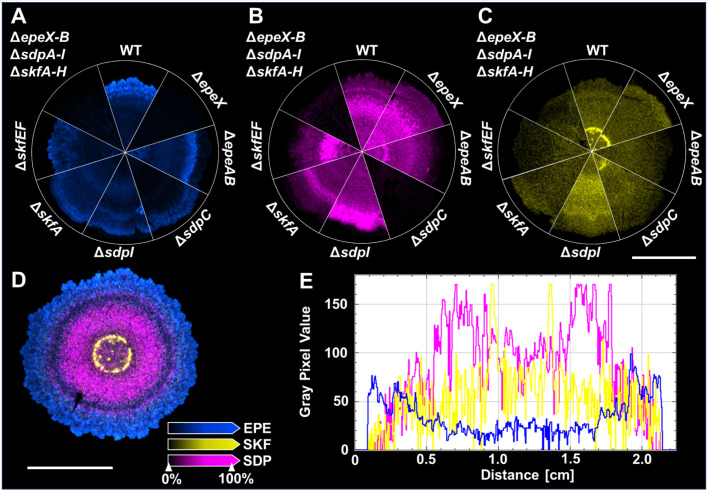
Overlay MALDI-MS images of cannibalistic toxins EPE, SDP, and SKF in WT and mutant *B. subtilis* biofilms. (**A–C**) Representative slices of the biofilm covering the center, inner, and outer zones, displaying the distribution of EPE (blue), SDP (magenta), and SKF (yellow) (from left to right) in the WT and cannibalistic mutant strains. (**D**) Distribution of EPE, SKF, and SDP across the whole colony of the WT. Scale bar indicates 1 cm. (**E**) A 2D-plot of toxin distribution across the whole WT biofilm conducted by linear pixel intensity measurement (gray pixel values).

Given the pronounced architectural changes observed in cannibalism mutants, we next examined whether these phenotypes correlated with the spatial distribution of the cannibalism toxins EPE, SDP, and SKF within colony biofilms. To this end, we employed matrix-assisted laser desorption ionization mass spectrometry imaging (MALDI-MSI) (52) to visualize toxin localization at a spatial resolution of approximately 50 µm. MALDI-MSI is a label-free technique that registers all generated ions simultaneously within a mass acquisition range based on their specific mass-to-charge (m/z) values, which, in turn, are defined by the molecular composition and type of charge-providing adduct ion. Colony biofilms were grown on mixed cellulose ester membranes and analyzed to detect EPE, SDP, and SKF based on their characteristic mass-to-charge ratios (see Materials and Methods; Fig. 2; Fig. S2 and S3). False-color images are used to illustrate the registration (and ultimately occurrence within laser-probed sampling pixel in a semi-quantitative fashion) of the three cannibalism toxins across mature 8-day-old biofilms of the WT and all cannibalistic mutant strains. All colony biofilms investigated exhibited radial symmetry with regard to their morphologies and peptide toxin production, allowing representative colony sectors to be compared across strains (Fig. 2; Fig. S3). In WT biofilms, EPE (depicted in blue) was predominantly localized to the outer zone of the colony (Fig. 2A and D), whereas SDP (depicted in magenta) was enriched in the inner zone (Fig. 2B and D). SKF (yellow) exhibited a distinct distribution pattern, forming a prominent ring at the interface between the central and inner zones, with weaker signals detected elsewhere (Fig. 2C and D). Quantitative analysis of pixel intensity profiles confirmed that the three toxins collectively spanned the entire colony area, each occupying a preferred spatial niche (Fig. 2E).

Deletion of individual toxin-encoding genes altered these distribution patterns in a toxin-specific manner. As expected, EPE was absent from Δ*epeX* and ΔΔΔ biofilms (Fig. 2; Fig. S3), but SKF was strongly detected in the outer zone, where EPE was “originally” located in the WT. In the absence of SKF (Δ*skfA*), the characteristic outer-zone enrichment of EPE was lost, while SDP distribution became concentrated at the boundary between the inner and outer zones. Conversely, the absence of SDP (Δ*sdpC*) resulted in a modest accentuation of EPE signal in the outer zone.

Striking redistribution effects were observed in autoimmunity-deficient mutants. In the Δ*epeAB* mutant, SDP accumulated strongly at the central ring structure normally occupied by SKF in the WT, while the SKF signal itself was substantially reduced. In Δ*sdpI* biofilms, SDP shifted toward the outer zone, overlapping spatially with EPE (Fig. 2; Fig. S3). Notably, SKF was undetectable not only in Δ*skfA* mutants but also in Δ*skfEF* strains, indicating that SKF production depends on the presence of its autoimmunity system, which is not observable for EPE or SDP. Control analyses confirmed that weak background signals throughout the biofilm represent noise rather than residual toxin (Fig. S2).

Together, these data demonstrate that EPE, SDP, and SKF exhibit distinct but to some extent interdependent spatial distribution patterns within colony biofilms. Loss of individual toxins or autoimmunity systems leads to pronounced redistribution of the remaining toxins, suggesting a coordinated spatial organization of cannibalism toxin production across the biofilm.

### Expression of *epeX* correlates with the spatial localization of EPE activity

We had previously demonstrated that cannibalism toxins intrinsically trigger the CESR (33, 53). We therefore asked whether transcriptional reporters and CESR sensors could be used to relate toxin gene expression to spatial toxin localization during colony development. The promoters P*_liaI_*, P*_bceA_*, and P*_psdA_*, when fused to the *luxABCDE* reporter cassette, provide a highly specific, cannibalism-induced luminescent readout, with P*_liaI_* responding to EPE-stress, while P*_bceA_*/P*_psdA_* were triggered by both SDP and particularly by SKF, at least in liquid cultures of strains derived from the domesticated laboratory reference strain *B. subtilis* W168.

**Fig 3:**
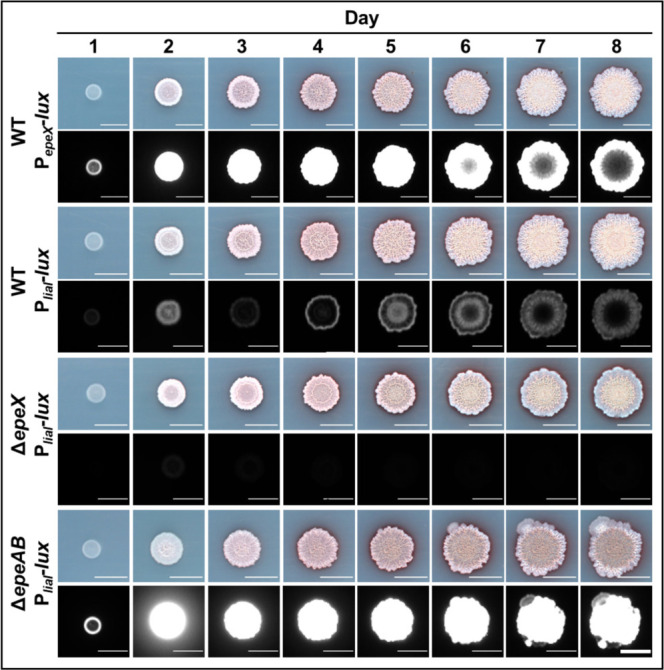
Activity of promoters of *epeX* and the corresponding stress response *liaI* fused to a *luxABCDE*-cassette in WT and mutant strains on MSgg agar. Colony expansion and luminescence signal distribution are shown over the course of 8 days. Scale bar indicates 1 cm.

We therefore monitored reporters for *epeX*, *sdpA*, and *skfA*, as well as CESR reporters (P*_liaI_*, P*_bceA_*, and P*_psdA_*) over 8 days of biofilm growth. Since the reporters previously validated in liquid cultures of domesticated strains failed to produce detectable signals for SDP- and SKF-related CES in DK1042-derived colonies, we exclusively focused on EPE-associated reporters (Fig. 3). The *epeX* promoter (P*_epeX_*) (Fig. S4Ci) exhibited strong activity throughout the whole developmental period, with early activity at the inoculation site (day 1) and later spreading across the colony (days 2–5). From day 6 onward, P*_epeX_* activity gradually diminished in the colony center and became confined to the outer zone. P*_liaI_* displayed a ring-like pattern during early development and became increasingly localized to the biofilm edge at later stages. By days 7 and 8, P*_liaI_* activity spatially overlapped with both P*_epeX_* activity and the MALDI-MSI-detected EPE signal.

Notably, the temporal offset between sustained *epeX* expression and delayed P*_liaI_* activation suggests that transcription of the *epeXEPAB* operon does not directly correspond to immediate release of active EPE. This observation indicates that EPE maturation or secretion is regulated beyond the transcriptional control. The interpretation is supported by the genetic architecture of the *epe* locus (Fig. S4Ci). While *epeX*, *epeE*, and *epeP* are transcribed from the P*_epeX_* promoter, a predicted stem-loop-forming terminator structure is located between *epeX* and *epeE*, indicating that expression of the three genes required for EPE biosynthesis (*epeXEP*) may require additional regulatory inputs beyond promoter activation. Such a terminator has been experimentally mapped and is further supported by transcriptome-wide tiling array analyses, which revealed growth-dependent changes in *epeEP* mRNA abundance that cannot be explained solely by transcriptional termination at the 3′ end of *epeB* (54–57).

Consistent with this interpretation, P*_liaI_* activity was completely absent in the Δ*epeX* mutant, confirming its specificity for EPE-induced stress (Fig. 3). The strongly elevated and broadly distributed P*_liaI_* activity in the autoimmunity mutant (Δ*epeAB*) demonstrates that EpeAB effectively counteracts EPE stress in colony biofilms.

These findings establish a spatial and temporal correlation between *epeX* expression, EPE release, and CESR activation, while suggesting post-transcriptional regulation of mature EPE production and release during biofilm development.

### EPE stress in autoimmunity mutants gives rise to suppressor mutants

During prolonged biofilm growth, we observed sporadic outgrowths at the periphery of Δ*epeAB* colonies, coinciding with regions of reduced P*_liaI_* activity (Fig. 1; Fig. S1; Fig. 3). Moreover, these “flares,” which were also detected in Δ*sdpI* mutant biofilms, remained white, suggesting a lack of sporulation, and remained stable upon re-streaking, suggesting the emergence of suppressor mutants under severe cannibalism stress (Fig. S4A).

One of these spontaneous mutants, which was isolated from the Δ*epeAB* background, was subsequently characterized by whole-genome sequencing of genomic DNA and luminescence-based analysis of P*_epeX_* and P*_liaI_* promoter activities in the corresponding strains (Fig. S4Cii and D). We identified a single nucleotide polymorphism (SNP) in the Spo0A binding site within the promoter region of *abrB* (Fig. S4B). Sequence analysis of the *abrB* promoter region from the other flares identified additional, but not identical, SNPs in the Spo0A consensus sequence (TGNCGAA) at genome positions 3,508,948 (G–A), 3,508,949 (T–C), 3,508,9454 (A–G), and 3,508,946 (C–T), indicating convergent evolution toward disruption of Spo0A-mediated *abrB* repression (20).

Functional analyses confirmed that these mutations resulted in elevated AbrB activity, leading to repression of *epeX* and *sdpC* expression (Fig. S4D). Consequently, P*_epeX_* activity was reduced approximately 10-fold, accompanied by a proportional decrease in EPE stress, as monitored via the P*_liaI_* CESR reporter (Fig. S4D).

AbrB is a global transition state regulator controlling over 250 genes, including those involved in matrix production, motility, quorum sensing, sporulation, and cannibalism (58–60). The recurrent emergence of suppressor mutations targeting the Spo0A–AbrB regulatory axis demonstrates that EPE-induced stress imposes strong selective pressure on biofilm populations, favoring regulatory rewiring that suppresses toxin production to restore population stability.

### Colony biopsy unravels cannibalism-dependent effects on spatial cell type distribution

**Fig 4:**
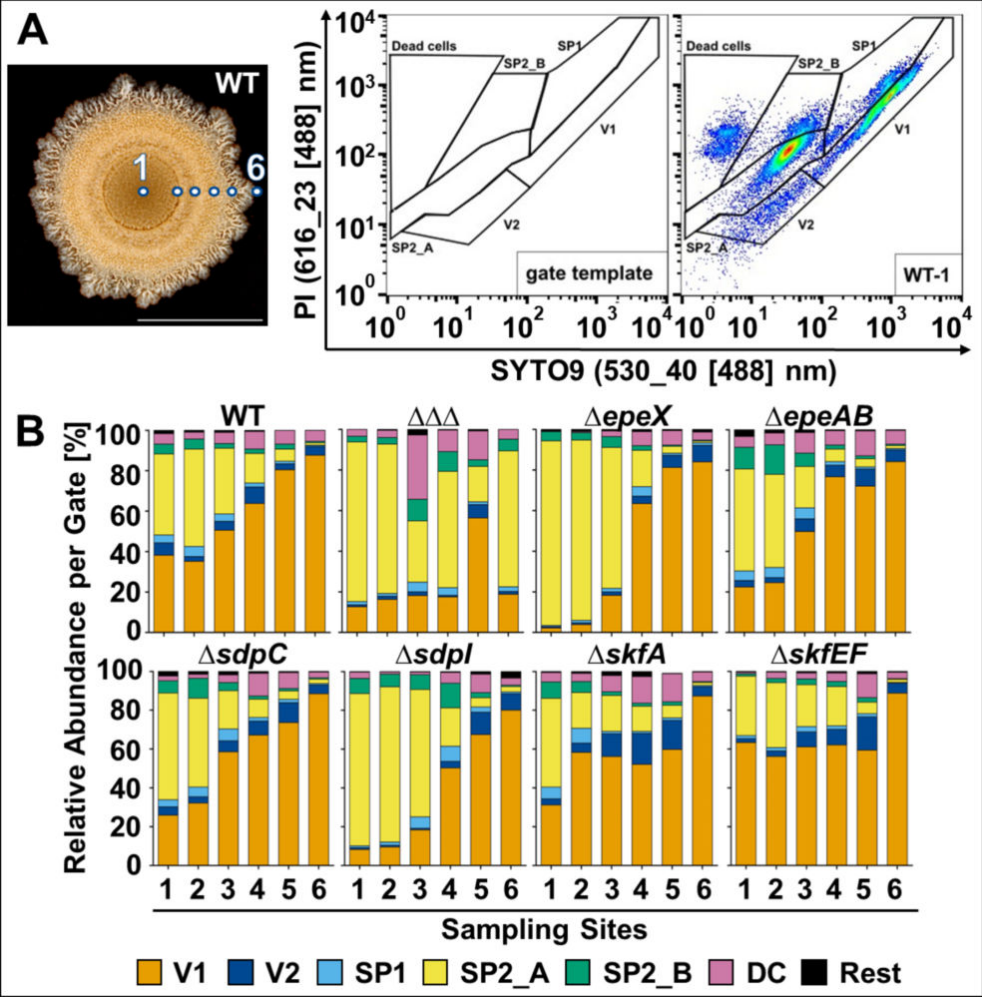
Distribution of live, dead, and sporulating cells across *B. subtilis* colonies in regard to cannibalism. (**A**) Sampling sites (1–6, from center to outer edge) across an exemplary WT colony (Scale bar indicates 1 cm) are shown, as well as the gate template for analysis and sorting together with an exemplary data set of flow cytometric measurement of cells present in sampling site 1 of the WT. The PI signal is plotted against the SYTO9 signal. (**B**) Overview of flow cytometric analysis results is summarized in bar graphs for each strain, where the relative abundance of cells per gate is plotted against sampling sites. V1 and V2 indicate vegetative cells, SP1–SP2_B represent different spore types, and DC indicates dead cells.

To determine whether the architectural and toxin-distribution phenotypes were accompanied by changes in colony composition, we analyzed spatial cell type distributions using colony biopsy combined with flow cytometry and cytometric fingerprinting (61). Fully developed 8-day-old biofilms were sampled at six defined positions spanning the colony center, inner zone, and outer zone (Fig. 4A; Information S1).

Each sample was then analyzed for the fractions of spores, live and dead cells, as well as cell cycle states to assess population heterogeneity (Information S3 [Fig. 1 through 3]). Both intrinsic parameters, such as scattering, and extrinsic parameters, such as fluorescence, were measured using flow cytometry. Living cells were differentiated from dead cells by combining staining with SYTO9 (a green-fluorescent, membrane-permeant nucleic acid stain) and propidium iodide (PI, which indicates compromised cell membranes and cell walls) (Fig. 4; Information S1 and S2; Information S3 [Fig. 2]). In addition to the SYTO9/PI staining, cell cycle states were assessed using 4′,6-di-amidino-2-phenylindole (DAPI), a dye that intercalates into AT-rich regions of double-stranded DNA. The resulting cell subsets are displayed as 2D plots, with cell gates defined for fluorescence-activated cell sorting (FACS) (Information S3 [Fig. 1 and 3]). Based on these gates, we analyzed the changes in cell type distribution between the WT and cannibalism mutant strains.

Six distinct subpopulations could be identified within *B. subtilis* biofilms, based on SYTO9-/PI-staining: three spore types (SP1, SP2_A, and SP2_B), two vegetative cell types (V1 and V2), and dead cells (DC and SI2). Dead cells exhibited only red PI fluorescence, while spores showed low to medium SYTO9 fluorescence compared to vegetative cells, which appeared in the rightmost gates of the 2D plots (Fig. 4A; Information S3 [Fig. 1]). In WT biofilms, spores were enriched in the colony center and adjacent inner zone, while vegetative cells predominated toward the periphery. Specifically, spores accounted for 48.9%, 58.1%, and 38.5 of cells at sampling sites 1–3, respectively, but declined to 18.7% at site 4 and to ~2.5% at the outermost sites, where vegetative cells comprised ~92% of the population (Fig. 4B; Information S3 [Fig. 1]). Dead cells were present at low levels throughout the colony (~5 at the periphery). This spatial pattern was confirmed by DAPI-based analyses (Information S3 [Fig. 1]), showing an excess of dormant spores and sporulating cells found in the center of the colony (53.69% at sampling site 1; Information S3 [Fig. 1]), and the proportion of spores also decreased toward the outer zone of the biofilm.

Deletion of individual toxin genes affected cell type distribution in a toxin-specific manner. The Δ*epeX* and Δ*sdpC* mutants exhibited a marked increase in spore abundance in central regions (sampling sites 1–2), whereas the Δ*skfA* mutant closely resembled the WT, as observed in both the SYTO9/PI and the DAPI-staining analyses (Fig. 4B; Information S3 [Fig. 1 to 3]; Fig. S1 to S3). The increased spore abundance, particularly in the center and adjacent regions of the inner zone, may explain the red coloration observed in the *epeX* mutant. Previous studies have shown that sites of sporulation tend to exhibit darker biofilm coloration (62, 63). The rise in spore numbers in toxin deletion strains is consistent with earlier observations (26). Autoimmunity mutants displayed more complex responses: despite elevated CESR activity, neither Δ*epeAB* nor Δ*sdpI* biofilms showed a substantial increase in overall dead cell abundance (Information S3 [Fig. 2 and 3]; Fig. S2 and S3). While dead cells constituted 5.6% of the WT population, this value increased only modestly in Δ*epeAB* biofilms (8.4%) and was reduced in Δ*sdpI* (3.8%) and Δ*skfEF* (4.3%) colonies.

The most pronounced alterations were observed in the hypo-cannibalistic mutant (ΔΔΔ). In this strain, spores were abundant across all sampling sites, including the outer zone, resulting in a near-complete loss of spatial zoning. Overall, ΔΔΔ colonies contained ~62% spores and only ~25 vegetative cells, compared to ~29% spores and ~64% vegetative cells in the WT. Comparable results were obtained in the DAPI analyses (Information S3, Fig. 1 and 3; Fig. S1 and S3). Although the total fraction of dead cells increased only slightly (+0.94% relative to WT), their spatial distribution shifted markedly, with elevated dead cell frequencies at sampling sites 3–5 (31.6%, 10.6%, and 14.2%, respectively), corresponding to regions of excessive wrinkling and structural deformation (Fig. 4B; Fig. 1; Fig. S1).

Taken together, these data demonstrate that cannibalism toxins are essential for maintaining the spatial zoning of cell types within colony biofilms. Loss of cannibalism disrupts the balance between vegetative growth and sporulation, leading to homogeneous, hyper-sporulating colonies with severely altered architecture.

## DISCUSSION

In this study, we investigated the spatial distribution of the cannibalism toxins EPE, SDP, and SKF across differentiated *B. subtilis* colony biofilms and analyzed how their distribution relates to colony architecture, cellular differentiation, and sporulation. By combining MALDI-MS imaging with phenotypic analyses, promoter activity measurements, and colony biopsy-based flow cytometry, we put cannibalism toxin production in a spatial and functional context within multicellular bacterial colonies.

Advanced MALDI-MS imaging enabled visualization of the two-dimensional distribution of EPE, SDP, and SKF across whole colony biofilms and revealed distinct yet partially overlapping localization patterns for each toxin. SKF was predominantly localized in the biofilm center, whereas SDP was mostly enriched in inner biofilm regions (Fig. 2; Fig. S3). Comparable spatial patterns for SDP and SKF have previously been reported for *B. subtilis* biofilms grown on MSgg agar (64), while discrepancies observed in other studies likely reflect differences in strain background and growth conditions, such as the use of domesticated *B. subtilis* PY79 on ISP2 medium, which yields less structured biofilms (65). Our work advances these earlier studies by integrating the epipeptide EPE into the spatial framework using a NCIB3610-derived background that retains key biofilm phenotypes. EPE was predominantly detected in the outer, actively growing zone of the biofilm (Fig. 2; Fig. S3), overlapping with regions of colony expansion (66). This localization is consistent with EPE’s established role as a competition determinant and antimicrobial peptide that induces membrane perturbations and cell envelope stress (32, 38, 67, 68). The spatial overlap between EPE abundance, regions with increased wrinkle formation, areas of accumulated cell envelope stress, and partial increases in propidium iodide-positive cells support a link between EPE production, membrane damage, and localized cell death (Fig. 4; Information S1 and S3).

MALDI-MS imaging also revealed pronounced interdependencies in toxin localization. Redistribution of SKF and SDP in strains lacking EPE production or autoimmunity (Fig. 2; Fig. S3) indicates that cannibalism toxins are not deployed independently but are spatially coordinated and depend on the presence of each other and their immunity systems. Classical models describe cannibalism toxins as individual effectors with defined roles in sporulation delay (26, 30). In contrast, our data support a model in which cannibalism functions as an integrated differentiation module, with coordinated toxin deployment contributing to biofilm organization (6, 69).

The MALDI-MSI data reveal that SKF is predominantly localized to the biofilm center with only minor presence at the periphery (Fig. S3). Preliminary three-dimensional MALDI-MSI data obtained from sagittal cross sections through the biofilms show SKF enrichment at the biofilm–agar interface, that is, within the pores of the mixed cellulose ester membrane used for cultivation (K. Dreisewerd, personal communication) (70). This finding demonstrates the considerable potential of resolving toxin distribution in three dimensions. Moreover, it highlights the need for continued advancement in the field, notwithstanding the substantial progress in methodology and technology that has been achieved recently (52, 71–73). Also, other studies found that the distribution of SKF is localized at the biofilm boundary and is probably dependent on surfactin production, as *srfAC* mutants are deficient in SKF production (74). Given the established role of surfactin in reducing surface tension and facilitating colony spreading (75–78), the co-occurrence of SKF and surfactin raises the hypothesis that SKF contributes to surface-associated or mechanically relevant processes during biofilm growth rather than functioning primarily as a killing factor under these conditions. This interpretation of SKF function would also fit the lack of antimicrobial property noted before (65), despite its name.

It is important to note that the limited contribution of SKF observed in this study may reflect the defined laboratory conditions used here. Growing a pure, isogenic biofilm on agar surfaces still represents a laboratory setup that can capture only a small subset of the environmental parameters encountered by *B. subtilis* in its natural habitat (79, 80). Under fluctuating or more complex environmental conditions, SKF may still play a meaningful role in modulating sporulation timing or colony morphology, potentially alongside other developmental or stress-response pathways.

Beyond their spatial distribution, our results reveal a strong connection between cannibalism, toxin production, and colony architecture. Pronounced phenotypic effects were observed primarily in strains lacking autoimmunity to EPE or SDP, as well as in the hypo-cannibalistic mutant (ΔΔΔ), whereas single toxin deletions had comparatively minor effects on colony morphology (Fig. 1; Fig. 3; Fig. S1). This indicates that, next to toxin production *per se*, toxicity control mediated through autoimmunity is critical for maintaining architectural stability. Mechanical deformation and vertical buckling of biofilms have previously been linked to nutrient limitation, growth arrest, and sporulation onset (43, 49, 81–84). In this context, our data suggest that cannibalism toxins restrict excessive vertical wrinkling while promoting lateral expansion, thereby maintaining access to nutrients at the biofilm–substrate interface. Vertical growth displaces cells away from nutrient sources and favors sporulation (43), whereas lateral expansion increases the surface area in contact with the substratum (here: agar) and improves nutrient access, and hence, expansion. Occurrence of cell death in the colony biofilm correlates with regions of EPE and SDP, but not SKF, production rather than with sporulation itself, as dead cell abundance slightly increases in toxin-rich regions where spore numbers remain low (Fig. 2; Fig. 4; Information S3). These observations support a model in which cannibalism-driven PCD contributes to architectural patterning rather than acting solely as a nutrient-recycling mechanism. Recent systematic time-lapse microscopy and quantitative analyses have provided key mechanistic insight into how colony biofilm architecture emerges from coordinated growth and matrix production (81, 85–88). In particular, Porter and colleagues demonstrated that radial expansion of *B. subtilis* colony biofilms is driven primarily by cell division at the periphery and that extracellular matrix components act in an obligate synergistic manner to provide mechanical stability, enable substrate deformation, and support complex three-dimensional structures (85). These findings reinforce the view that biofilm morphology is an active, regulated outcome of growth dynamics and mechanical interactions rather than a passive consequence of biomass accumulation. Within this framework, our data suggest that cannibalism toxins represent an important control layer that modulates the balance between vertical buckling and lateral expansion, thereby indirectly orchestrating nutrient access and developmental trajectories such as sporulation. Since cannibalism toxins are unlikely to act as structural components of the extracellular matrix, their impact on biofilm architecture appears to rather arise indirectly through regulatory and physiological effects on growth dynamics, differentiation, and localized cell death, which will require further investigations.

The architecture of multicellular colony biofilms is characterized by a spatiotemporal controlled phenotypic diversification of different cell types—a result of the regulatory cascades governing differentiation in response to external stimuli such as nutrient and oxygen availability in combination with the underlying stochastic gene expression "noise" (89–93). In this study, flow cytometry revealed, next to others, multiple spore-associated subpopulations distinguishable by SYTO9/PI and DAPI staining, indicating heterogeneity in spore states within colony biofilms (Fig. 4; Information S1 [Fig. 1]). Such heterogeneity is increasingly recognized as a fundamental feature of *B. subtilis* sporulation and has been linked to trade-offs between spore quantity, quality, and stress resistance (94–96). *B. subtilis* populations deliberately generate spores with variable resistance properties depending on environmental conditions and developmental history, thereby favoring a diversified survival strategy (94, 97). In this context, the altered sporulation patterns observed in toxin-deficient, autoimmunity-deficient, and hypo-cannibalistic mutants may not only reflect changes in sporulation timing or abundance but could also indicate shifts in spore maturation or robustness driven by altered stress landscapes and resource allocation (98–100). However, resolving whether cannibalism directly influences spore quality will require further research.

**Fig 5:**
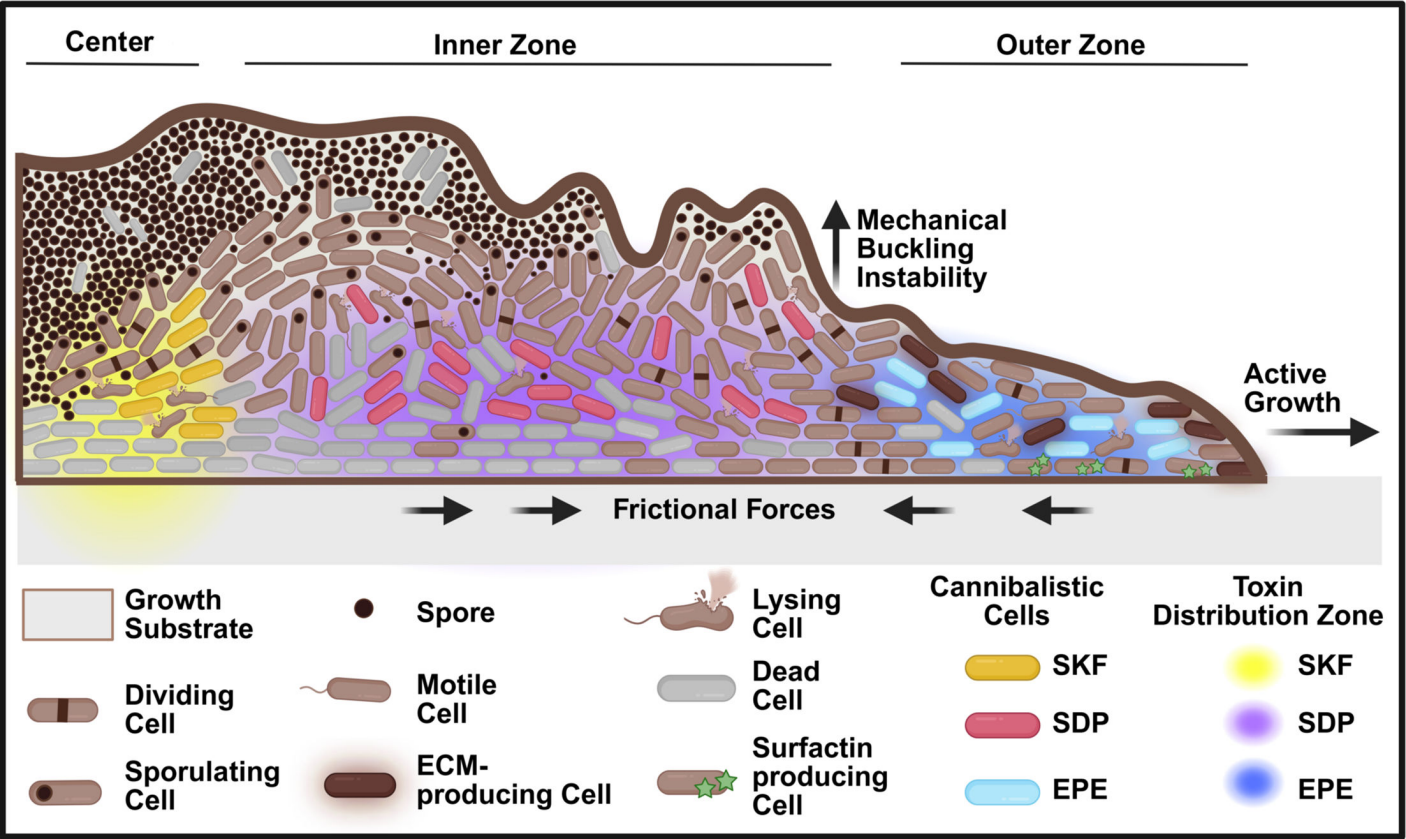
Schematic illustration of a *B. subtilis* biofilm cross section illustrating the distribution of differentiated cell types and cannibalistic toxins SKF, SDP, and EPE across the biofilm. The growth substrate beneath the biofilm relates to this study, where we used MSgg agar (6, 82, 103, 104). Created with BioRender.com.

Rather than forming rigidly separated cell types, recent work has demonstrated that differentiation strategies in *B. subtilis* biofilms are spatially patterned yet transcriptionally overlapping, resulting in extensive multicellular tasking within the population (6, 11, 43, 81, 101). Single-cell reporter analyses, flow cytometry, and multimodal imaging approaches have revealed that matrix production, cannibalism, and sporulation-associated gene expression frequently co-occur within individual cells, challenging the classical view of discrete, mutually exclusive cell types (6, 101, 102). Within this conceptual framework, cannibalism emerges as a differentiation strategy that delays commitment to sporulation by maintaining lateral colony expansion and suppressing excessive vertical buckling, thereby enhancing population-level adaptability in unpredictable environments (3). Our findings and the aspects discussed above lead us to suggest an updated model of cannibalism in *B. subtilis* multicellular life, as illustrated in Fig. 5.

While our study sheds important light on the structural role of cannibalism and its functional consequences for phenotypic heterogeneity within colony biofilms, it also underscores the importance of bacterial PCD for multicellular differentiation and tissue structuring. This connection, long established for higher eukaryotic tissues, has only recently emerged for bacteria, owing to technological advances that enable the resolution of bacterial macroscopic structures at or near single-cell resolution (51). Initial data indicate that localized PCD also seems to play a role in the multicellular differentiation of aerial hyphae in streptomycetes and fruiting body formation in myxobacteria (105, 106), which offers exciting new directions in uncovering new traits in bacterial multicellularity. Together, these findings highlight bacterial biofilms as dynamic, self-organizing tissues and underscore the need to further dissect the regulatory interplay between differentiation, metabolism, and mechanics in multicellular bacterial life.

## MATERIALS AND METHODS

### Reagents

Chemicals used in this study were obtained from Thermo Fisher Scientific (Waltham, MA, USA), Carl Roth GmbH & Co. KG (Karlsruhe, Germany), or Merck KGaA (Sigma Aldrich, Darmstadt, Germany). All enzymes (restriction endonucleases, ligases, and polymerases for PCR) originated from New England Biolabs (Ipswich, MA, USA), and general cloning procedures were performed according to the recommended protocols. PCR purifications and plasmid preparations were undertaken using the corresponding kits from Süd-Laborbedarf GmbH (Gauting, Germany).

### DNA isolation

#### Isolation of genomic DNA from *B. subtilis* for transformation

In total, 800 µL of overnight culture was transferred into a 2 mL Eppendorf tube and mixed with an equal volume of saline-citrate (SC) buffer (NaCl 0.15 M, sodium citrate 0.01 M, pH 7.0). Cells were then harvested by centrifugation at 14,000 × *g* for 1 min and afterward resuspended in 1 mL SC buffer; 20 µL lysozyme (15 mg/mL) was added, and the mix was incubated at 37°C for 15 min. Afterward, 800 µL of 5 M NaCl and 200 µL H_2_O were added, followed by filtration of the DNA solution through a 0.45 µm syringe filter (Sarstedt AG & Co. KG, Nümbrecht, Germany). The DNA solution was directly used for the transformation of *B. subtilis* or otherwise stored for later use at −20°C.

#### Isolation of total DNA from *B. subtilis*

For isolation and rapid purification of highly pure genomic DNA of *B. subtilis*, the NucleoSpin Microbial DNA kit (Macherey-Nagel GmbH & Co. KG*,* Düren, Germany) Kit was used, following the manufacturer’s instructions.

### Strain construction

All DNA oligonucleotides and bacterial strains used in this study are listed in Tables S1 and S2, respectively. The synthesis of oligonucleotides was performed by Microsynth AG (Balgach, Switzerland). DNA sequencing was performed by Eurofins Genomics Europe Shared Services GmbH (Ebersberg, Germany).

The *epeXEPAB* deletions in *B. subtilis* were constructed by allelic replacement mutagenesis using long flanking homology, PCR (LFH-PCR). The technique is derived from a published procedure (107) and was performed as described previously (108, 109). The kanamycin resistance cassette was amplified from the suitable template vector pDG780 (110), using TM0137/TM0138. Two primer pairs were designed to amplify ∼1,000 bp DNA fragments flanking the region to be deleted at its 5′ and 3′ ends (TM7999/TM8000 and TM8001/TM8002). The resulting fragments are here called the “up” and “do” fragments. The 3′ end of the up fragment, as well as the 5′ end of the do fragment, extended into the gene(s) to be deleted in a way that all expression signals of genes up- and downstream of the targeted genes remained intact. Extensions of ∼25 nucleotides were added to the 5′ end of the up-reverse and the do-forward primers that were complementary (opposite strand and inverted sequence) to the 5′ and 3′ ends of the amplified resistance cassette. All obtained fragments were purified using the PCR purification kit from Süd-Laborbedarf GmbH (Gauting, Germany); 100–150 ng of the up and do fragments and 250–300 ng of the resistance cassette were used together with the specific up-forward and do-reverse primers at standard concentrations in a second PCR. In this reaction, the three fragments were joined by the 25-nucleotide overlapping complementary ends and simultaneously amplified by normal primer annealing. The PCR products were directly used to transform *B. subtilis*. Transformants were screened by colony PCR, using the up-forward primer with a reverse check primer annealing inside the resistance cassette (TM7999/TM0147). The integrity of the regions flanking the integrated resistance cassette and the cassette itself was verified by sequencing PCR products amplified from chromosomal DNA of the resulting mutants.

All other DK1042 mutants were constructed through re-transformation of the corresponding *B. subtilis* 168 mutants. All source strains were checked by sequencing, according to the description above. Transformation of *B. subtilis* was performed as described previously (111), adding 100 µL of chromosomal donor-DNA into the mix. Transformants were screened by colony PCR (for oligonucleotides used, see Table S1).

### Growth conditions

For routine growth of *B. subtilis*, lysogeny broth liquid media was made using the following recipe: 1% (wt/vol) Bacto-peptone, 1% (wt/vol) NaCl, and 0.5% (wt/vol) yeast extract. For solid plates, LB broth was supplemented with 1.5% (wt/vol) agar. LB media were sterilized by autoclaving. For the transformation of *B. subtilis*, chemically defined medium MNGE (88.2% 1× MN medium comprising 1.36% [wt/vol] dipotassium phosphate × 3 H_2_O, 0.6% [wt/vol] monopotassium phosphate, 0.1% [wt/vol] sodium citrate × H_2_O, 1.9% glucose, 0.19% potassium glutamate, 0.001% [wt/vol] ammonium ferric citrate, 0.005% [wt/vol] tryptophan, and 0.035% [wt/vol] magnesium sulfate) was used. Single isolate biofilm assays were conducted using MSgg medium. MSgg was made by first preparing a base medium, consisting of 5 mM potassium phosphate, 100 mM MOPS at pH 7.0, supplemented with 1.5% (wt/vol) agar. The base medium was autoclaved and cooled to 55°C. The base medium was supplemented with 2 mM MgCl_2_, 700 µM CaCl_2_, 50 µM FeCl_3_, 50 µM MnCl_2_, 1 µM ZnCl_2_, 2 µM thiamine, 0.5% (vol/vol) glycerol, and 0.5% (wt/vol) glutamic acid. A volume of 25 mL of melted MSgg medium was added to each 10 cm square Petri dish (Sarstedt AG & Co. KG, Nümbrecht, Germany) and solidified at room temperature. The surface of the solid plates was dried for 45 min in a laminar flow cabinet before use in experiments.

*B. subtilis* cells carrying a resistance marker were selected using spectinomycin (100 μg/mL), tetracycline (12.5 µg/mL), kanamycin (10 μg/mL), or erythromycin (1 μg/mL) combined with lincomycin (25 μg/mL) for MLS (macrolide, lincosamide, and streptogramin B).

### Biofilm formation assay

For growing biofilms, overnight cultures in LB medium with selective antibiotics were diluted to an OD_600_ of 0.1 in 1 mL LB medium. Carefully, 5 µL of the cell suspension were spotted on MSgg agar plates (4), which were dried for 45 min beforehand. For MALDI-MS-Imaging (MALDI-MSI) analysis and flow cytometry, 5 µL of the cell suspension were spotted onto a mixed cellulose esters filter membrane with a 0.22 µm average pore size (EZGSWG474, Merck Millipore, Darmstadt, Germany), which were placed on MSgg agar. Agar plates were incubated at 25°C. Pictures were taken daily using a P.CAM360 (TU Dresden, Dresden, Germany) or a binocular microscope (WILD M3Z, Leica Microsystems GmbH, Wetzlar, Germany) equipped with a ProgRes “SpeedXT core5” camera (Jenoptik AG, Jena, Germany, and Capture Pro software) over the course of 8 days. Assays were performed in biological duplicates and technical triplicates.

### Luciferase assay

For luciferase assays on MSgg agar plates, biofilms were grown as previously described above in “Biofilm formation assay.” Luminescence was detected with a Fusion FX system (Vilber Lourmat GmbH, Eberhardzell, Germany). The exposure times for the luminescence reporters were as follows: P*_epeX_* 5 s; P*_sdpA_* and P*_skfA_* 10 s; and P*_bceA_*, P*_psdA_*, P*_liaI_*, and P*_empty_* 2 min. Assays were performed in biological duplicates and technical triplicates.

### MALDI-MS imaging

Biofilms were grown following the procedure described in “Biofilm formation assay” above. The membranes were used to ensure easy detachment of the biofilms from the agar and to simplify subsequent inactivation as described by Brockmann and colleagues for steam inactivation of non-sporulating bacteria (52). Because of the heat resistance of the *B. subtilis* spores, bacterial biofilms were, in our case, cut out and wetted with a solution of 10% (vol/vol) formaldehyde (Carl Roth GmbH & Co. KG, Karlsruhe, Germany) in water. After 30 min, fixation was complete, and the biofilms were washed two times with Milli-Q water (Merck KGaA, Darmstadt, Germany) and then glued together with the membrane on a microscope slide using super glue (UHU GmbH & Co KG, Bühl, Germany). A VS200 slide scanner with a 4× objective (Evident GmbH, Hamburg, Germany) and ORCA-Fusion camera (C14440 20UP, Hamamatsu Photonics, Hamamatsu City, Japan) was used for reflected light microscopy of biofilms. For microscope image visualization, OlyVia (version 3.4.1, Evident*,* Hamburg, Germany) was used. A solution of 7 mg/mL 2,5-dihydroxyacetophenone (Merck KgaA, Darmstadt, Germany) dissolved in 75% acetonitrile (Carl Roth GmbH & Co. KG, Karlsruhe, Germany), 10% methanol (Carl Roth GmbH & Co. KG, Karlsruhe, Germany), 10% trifluoroacetic acid (Carl Roth GmbH & Co. KG, Karlsruhe, Germany), and 5% Milli-Q water was sprayed on the biofilm as MALDI matrix using a SunCollect pneumatic spray robot (SunChrom, Friedrichsdorf, Germany). The matrix was applied within 22 spraying cycles at a nitrogen back pressure of 2.5 bar in a meandering pattern. The flow rate followed a gradient and started at 15 µL/min in the first cycle and increased to 20 µL/min and 30 µL/min for the second and third spraying cycles, respectively. From the fourth spraying cycle onward, the flow rate was 50 µL/min until the end. The spray nozzle moved with a velocity of 700 mm/min and a line distance of 2 mm. The distance between the spray nozzle and the sample surface was set to 44 mm.

All MALDI-MSI measurements were conducted with a Bruker timsTOF fleX MALDI-2 QTOF mass spectrometer (Bruker Daltonics GmbH & Co. KG, Bremen, Germany), equipped with a Smartbeam 3D laser emitting at 355 nm and providing a focal spot size of 5 µm diameter. For all imaging experiments, the resulting field size was set to 50 µm using the beam scan function and the “M5 small” setting; 200 laser shots were applied per 50 µm-wide pixels at a laser pulse frequency of 1 kHz. The nitrogen buffer gas pressure in the MALDI ion source of the hybrid orthogonal extracting time-of-flight mass spectrometer (QTOF) was set to 2.5 mbar. With the analytical focus on the detection of the peptide toxins, all herein reported MSI data were acquired in the “high sensitivity detection” mode and the use of an *m*/*z* data acquisition range of 1,000–4,500 in positive ion mode. With a focus on the detection of the peptide toxins, the optional MALDI-2-postionization mode of the instrument was not used. MSI data were processed using SCiLS Lab MVS (vs. 2024a Pro, Bruker Daltonics GmbH & Co. KG, Bremen, Germany), and ion images were visualized as false color imaging using the same software. MSI experiments were performed in biological and technical duplicates.

### Microbial flow cytometry and cytometric fingerprinting

Flow cytometric analysis followed the procedure described by Abbaszade and colleagues (61). In short, a BD Influx v7 Cell Sorter (Becton Dickinson, Franklin Lakes, NJ, USA) equipped with a stream-in-air nozzle of 70 µm was used. The blue 488 nm Sapphire OPS laser (400 mW) was used for measurement of the forward scatter (FSC, 488/10; PMT1; related to cell size), the side scatter (SSC, trigger signal, 488/10; PMT2; related to cell density), the SYTO9 fluorescence (530/40; PMT3), and the PI fluorescence (616/23; PMT5). The UV laser 355 nm Genesis OPS laser (100 mW) was used to measure DAPI fluorescence (460/50; PMT9). The fluidics ran at 33 psi through a 70 μm nozzle, and the cells were detected equivalent to an event rate of 2,500–3,000 events per second. The calibration of the cytometer was performed in the linear range by using 1 μm-sized blue fluorescent FluoSpheres (Ref. F-8815, Molecular Probes, Eugene, OR, USA), and 2 μm YG fluorescent FluoSpheres (Ref. F-8827, Thermo Fisher Scientific, Waltham, MA, USA). For calibration in the logarithmic range, 0.5 μm and 1 µm YG fluorescent FluoSpheres (Ref. F-8813 and F-13081, Thermo Fisher Scientific, Waltham, MA, USA) were used. As illustrated in Fig. 4A, biopsy samples were taken from one biological replicate using a 200 µL pipette tip from six different positions, covering the biofilm from the center to the outer edge. Due to the low cell count, biopsy samples were not OD-adjusted during cell handling. For analysis of population heterogeneity related to cell cycle states, 4′,6-di-amidino-2-phenyl-indole (DAPI) staining was performed as described in SI4 and reference (112). For discrimination between live and compromised cells, the combination of SYTO9 and propidium iodide (PI) staining was used as described in SI4 and reference (61). For each bacterial strain, 50,000 cells were measured flow cytometrically and visualized in 2D plots using the program FlowJo 10.0.8.r1 (FlowJo, Becton Dickinson, Franklin Lakes, NJ, USA). Cell gate setting and the creation of the gate template was done as suggested in a previous study (61). FACS of different cell types for fluorescence microscopy was done using the gates from the gate template. For each gate, 50,000–200,000 cells were sorted. Data analysis followed the description published elsewhere (61).

### Fluorescence microscopy

Agarose pads were used as a measure to immobilize the spores in preparation for microscopy. Therefore, 1% UltraPure Agarose (Life Technologies GmbH, Darmstadt, Germany) was added to a rubber mold stuck to a slide and covered with a second slide until hardened. Size measurements were 20 mm in diameter and about 1 mm in depth; 2 μL of cell suspension was added on top of the agarose pads and covered with a coverslip. The prepared slides were analyzed using a Zeiss AXIO Observer Z1 microscope, equipped with an Axiocam 503 monochrome camera (Carl Zeiss AG, Oberkochen, Germany). Upon use of an immersion objective PlanApo 63×/1.40 Oil Ph3 M27, NA 1.4 a magnification of 630× was achieved. Microscopy was performed using phase contrast (Ph3, light intensity: 4.4 V, exposure time: 1.59 s) and BFP channel (exposure time: 3.13 s for gates: c1n, c2n, c3n, csp1, sp1; 1.81 s for gates sp3, sp4). Excitation and emission wavelengths were 353 nm and 465 nm, respectively. Analysis of the microscopy images was performed using the software Fiji (“ImageJ”). Rectangles were cropped from the original image, representative of cells of each cell type.

### Whole genome sequencing and comparison

Genomic DNA was isolated with the NucleoSpin Microbial DNA Kit according to the manufacturer’s protocol. Whole genome sequencing was performed by the Microbial Functional Genomics Group of the Ludwig Maximilian University (LMU, Munich, Germany) using Sanger/Illumina 1.9 (paired end read) for analysis of the *epeAB* mutant flare (TMB6336).

Data analysis was performed using the open-source platform Galaxy EU (24.1) (usegalaxy.eu, Freiburg Galaxy Team). For TMB6336, the quality of the Illumina paired-end data was verified with “FastQC” (Galaxy v.0.11.8) (113). To automatize quality and adapter trimming, the sequencing data were analyzed with “Trim Galore!” (Galaxy v.0.4.3.1; threshold: 20; maximum error rate: 0.1; minimum reads length: 20), and sequences bearing an average quality below the threshold value were subsequently removed using “Trimmomatic” (Galaxy v.0.36.5; Average quality required: 20; Number of bases to average across: 4) (114). High-quality paired-end data were then assembled as contigs using “Unicycler” (Galaxy v.0.4.8.0; minimum length of contigs: 100 bp) (115). Quality check of the assembled genome was performed using “Quast” (116–118). Subsequently, the genome assembly contigs were aligned with the genome of *B. subtilis* (ASM904v1) using the “RagTag” tool (reference-guided scaffolding of draft genomes; Galaxy Version 2.1.0+galaxy1) (119). For genome annotation, the software tool “Prokka” was used (120, 121). The assembled genome was aligned to the reference genome to find single-nucleotide polymorphisms (SNPs), insertions, and deletions using the “snippy” tool with the default parameters (Galaxy Version 3.2) (122). For visualization, the script “JBrowse genome” (Version 1.16.11) was used (123).

## Data Availability

Microscopic images and related MALDI-MSI data are available at https://omero-imaging.uni-muenster.de/openlink/rn_TTOF224VTTK2_3157_2026-05-27_16-56-04/. The sequencing data were deposited in the NCBI Sequence Read Archive (SRA) under accession number PRJNA1449522 (https://www.ncbi.nlm.nih.gov/sra/PRJNA1449522) and are publicly available to ensure data accessibility and reproducibility. Obtained flow cytometry data are stored in the Zenodo database under the following DOI: https://zenodo.org/records/14967544.

## SUPPLEMENTAL MATERIAL

Supplemental Material mbio.00525-26-s0001.docx Tables S1 and S2, Fig. S1 to S4, and Information S1 to S4.

ASM does not own the copyrights to Supplemental Material that may be linked to, or accessed through, an article. The authors have granted ASM a non-exclusive, world-wide license to publish the Supplemental Material files. Please contact the corresponding author directly for reuse.
