# Supplementary material for: Cannibalism shapes biofilm structure and composition in Bacillus subtilis
Source: mBio. 2026 Jun 15;17(7):e00525-26. doi: 10.1128/mbio.00525-26 (PMC13343845; doi:10.1128/mbio.00525-26)
Supplement: Supplemental Material — Tables S1 and S2, Fig. S1 to S4, and Information S1 to S4. [file mbio.00525-26-s0001.docx]

**Supplementary Information**

**for**

**Cannibalism Shapes Biofilm Structure and Composition in *Bacillus subtilis***

Lena Friebel^A^, Jan-Philipp Knepper^B^, Nathalie Becker^A,C*^, Gorkhmaz Abbaszade^D^, Kathrin Stückrath^D^, Jens Soltwisch^B^, Susann Müller^D^, Klaus Dreisewerd^B^, and Thorsten Mascher^A#^

^A^TUD Dresden University of Technology, Chair of General Microbiology

^B^University of Münster, Institute of Hygiene

^C*^University of Tübingen, Interfaculty Institute of Microbiology and Infection Medicine
 Tübingen

^D^Helmholtz Centre for Environmental Research-UFZ, Department of Applied Microbial Ecology

# [thorsten.mascher@tu-dresden.de](mailto:thorsten.mascher@tu-dresden.de)

## **Table of Contents**

[Table of Contents 2](#_Toc222839200)

[Table S1 DNA oligonucleotides used in this study 3](#_Toc222839201)

[Table S2 Bacterial strains used in this study 5](#_Toc222839202)

[Figure S1 Growth of colonies of *B. subtilis* WT and cannibalism mutants 7](#_Toc222839203)

[Figure S2 MALDI-MS Imaging mass spectra. 8](#_Toc222839204)

[Figure S3 Overview MALDI-MS Imaging results 10](#_Toc222839205)

[Figure S4 Analysis of suppressor mutants arising in Δ*epeAB* colony biofilms. 11](#_Toc222839206)

[Supplementary Information 1: Testing viability of cells via triplicate analysis and cell sorting of *B. subtilis* wild type (WT) 13](#_Toc222839207)

[Supplementary Information 2: Back-gating procedure to verify gate affiliation in double stained 2D-dot plots 16](#_Toc222839208)

[Supplementary Information 3: Colony biopsy in combination with microbial flow cytometry and cytometric fingerprinting 18](#_Toc222839209)

[Supplementary Information 3 - Figure 1: Distribution of cell types across *B. subtilis* colonies regarding cannibalism after DAPI staining. 18](#_Toc222839210)

[Supplementary Information 3 – Figure 2: Overview of flow cytometric results for SYTO9/PI staining. 20](#_Toc222839211)

[Supplementary Information 3 – Figure 3: Overview of flow cytometric results for DAPI staining. 22](#_Toc222839212)

[Supplementary Information 4: Staining procedures for flow cytometry 23](#_Toc222839213)

[References 24](#_Toc222839214)

## Table S1 DNA oligonucleotides used in this study

| **Name** | **Sequence 5’ -3’** | **Source/ Reference** |
| --- | --- | --- |
| TM2262 | GAGCGTAGCGAAAAATCC | pBS3Cluxcheckfwd |
| TM2263 | GAAATGATGCTCCAGTAACC | pBS3Cluxcheckrev |
| TM2505 | CTGATTGGCATGGCGATTGC | pBS3Clux sacA front check fwd |
| TM2506 | ACAGCTCCAGATCCTCTACG | pBS3Clux sacA front check rev |
| TM2507 | GTCGCTACCATTACCAGTTG | pBS3Clux sacA back check fwd |
| TM2508 | TCCAAACATTCCGGTGTTATC | pBS3Clux sacA back check rev |
| TM0148 | GTTTTGGTCGTAGAGCACACGG | mls-check rev |
| TM0149 | CGTATGTATTCAAATATATCCTCCTCAC | spec-check rev |
| TM0147 | CTGCCTCCTCATCCTCTTCATCC | kan-check rev |
| TM0137 | CAGCGAACCATTTGAGGTGATAGG | kan-fwd |
| TM0138 | CGATACAAATTCCTCGTAGGCGCTCGG | kan-rev |
| TM2744 | TGGTGCGTTAGGGGTTATGATTGC | skf up fwd |
| TM3316 | GCTTCCCTAAGCTGTATTTGAACC | skfA do rev |
| TM2747 | GATTTGCTGCCGTTTTGGTAAGAC | skf do rev |
| TM3657 | CAGCAAGCATAATTTCAAATATCAG | sdpRI do rev LFH |
| TM7699 | TGGCACATACTATTCGCAAAA | sdpR_BKE_checkfwd |
| TM2748 | GAAGGTTATATTGACACCTATAATCC | sdpC up fwd |
| TM2751 | CAAATATCTAAATGTCTAAATGTTTTTTTGTAAAG | sdpC do rev |
| TM5426 | GTGATCAGCTTATCGGCTTG | yydF(epeX)-up-fwd |
| TM8140 | GGATAGAAGTAGTTTATTTTTTCTGC | oLF_epeX_rev |
| TM5430 | GAGGAATTTGACATTCCTTATG | yydI-up-fwd |
| TM8143 | CTAGCCATCTTTGCGCTC | oLF_epeA_rev |
| TM3324 | CATCGTTTTAGTAATGATCTGACC | skfEF do rev |
| TM3321 | CAGTACTTATTGGTACATAGCGG | skfEF up fwd |
| TM7766 | ATGTCAGCAAACATCGCAC | oLF_sdpABCRI up_fwd |
| TM7769 | ACTGTTCACCGGTGATTCC | oLF_sdpABCRI do_rev |
| TM3655 | GATGAATTGAAACAATCGATTGACAG | sdpR do rev LFH |
| TM7999 | CGGTGATCAGCTTATCG | oLF_EPE_upfwd |
| TM8000 | CCTATCACCTCAAATGGTTCGCTGATCCCTCCTCCTTTTC | oLF_EPE_uprev |
| TM8001 | CGAGCGCCTACGAGGAATTTGTATCGGCCTTTAATAAAGGGAGCTAC | oLF_EPE_dofwd |
| TM8002 | TACCAGCAAATCGCAAC | oLF_EPE_dorev |
| TM7503 | CCGCTCGGATAATAAATCGG | oNB_PabrB_seq_fwd |
| TM7504 | CGATAGGAATAACTACACGTCC | oNB_PabrB_seq_rev |

## Table S2 Bacterial strains used in this study

| **Name** | **Genotype and Relevant Features*** | **Source / Reference** |
| --- | --- | --- |
| DK1042 (WT) | *B. subtilis* NCIB3610 *comI^Q12L^* | [[1]](#_CTVL001a76de9c4e93346f6b79d1da774e4e74e) |
| TMB7067 | *B. subtilis* DK1042 *epeX::mls* | This study |
| TMB6126 | *B. subtilis* DK1042 *epeAB::spec* | This study |
| TMB6336 | *B. subtilis* DK1042 *epeAB::spec* “flare” mutation | This study |
| TMB6363 | *B. subtilis* DK1042 *epeAB::spec* “flare” mutation | This study |
| TMB6364 | *B. subtilis* DK1042 *epeAB::spec* “flare” mutation | This study |
| TMB6365 | *B. subtilis* DK1042 *epeAB::spec* “flare” mutation | This study |
| TMB6366 | *B. subtilis* DK1042 *epeAB::spec* “flare” mutation | This study |
| TMB7060 | *B. subtilis* DK1042 *sdpC::kan* | This study |
| TMB7056 | *B. subtilis* DK1042 *sdpI::mls* | This study |
| TMB7069 | *B. subtilis* DK1042 *skfA::mls* | This study |
| TMB6978 | *B. subtilis* DK1042 *skfEF::mls* | This study |
| TMB6980 | *B. subtilis* DK1042 *epeXEPAB::kan, skfABCEFGH::spec, sdpABC-sdpRI::tet* (ΔΔΔ) | This study |
| TMB6136 | *B. subtilis* DK1042 *sacA::*pBS3C*lux*-P*_liaI_* | This study |
| TMB6170 | *B. subtilis* DK1042 *sacA::*pBS3C*lux*-P*_epeX_* | This study |
| TMB6171 | *B. subtilis* DK1042 *sacA::cm* pBS3C*lux*-P*_empty_* | This study |
| TMB6217 | *B. subtilis* DK1042 *sacA::*pBS3C*lux*-P*_liaI_*, *epeAB*::*spec* | This study |
| TMB6220 | *B. subtilis* DK1042 *sacA*::pBS3C*lux*-P*_liaI_*_,_ *epeX*::*mls* | This study |
| TMB6351 | *B. subtilis* DK1042 *sacA*::pBS3C*lux*-P*_sdpA_* | This study |
| TMB7278 | *B. subtilis* DK1042 *sacA*::pBS3C*lux*-P*_sdpA_*, *sdpI*::*mls* | This study |
| TMB6733 | *B. subtilis* DK1042 *sacA*::pBS3C*lux*-P*_skfA_* | This study |
| TMB7277 | *B. subtilis* DK1042 *sacA*::pBS3C*lux*-P*_skfA_, skfEF::mls* | This study |
| TMB7279 | *B. subtilis* DK1042 *sacA*::pBS3C*lux*-P*_bceA_* | This study |
| TMB7280 | *B. subtilis* DK1042 *sacA*::pBS3C*lux*-P*_bceA_*, *sdpI*::*mls* | This study |
| TMB7281 | *B. subtilis* DK1042 *sacA*::pBS3C*lux*-P*_bceA_*, *skfEF::mls* | This study |
| TMB7282 | *B. subtilis* DK1042 *sacA*::pBS3C*lux*-P*_psdA_* | This study |
| TMB7283 | *B. subtilis* DK1042 *sacA*::pBS3C*lux*-P*_psdA_*, *sdpI*::*mls* | This study |
| TMB7284 | *B. subtilis* DK1042 *sacA*::pBS3C*lux*-P*_psdA_*, *skfEF::mls* | This study |
| BKE40180 | *B. subtilis* 168 *yydF(epeX)*::*mls* | [[2]](#_CTVL001fedb873be4d642a1bb56f731a8b87906) |
| HB6267 | *B. subtilis* CU1065 *yydIJ(epeAB)::spec* | [[3]](#_CTVL0016dd95c45955f4b539714e80cb8318ed3) |
| TMB1768 | *B. subtilis* 168 *sdpC::kan* | [[4]](#_CTVL0017d8b01ce1e584228933560ec7bdb61c1) |
| BKE33780 | *B. subtilis* 168 *sdpI::mls* | [[2]](#_CTVL001fedb873be4d642a1bb56f731a8b87906) |
| BKE01910 | *B. subtilis* 168 *skfA::mls* | [[2]](#_CTVL001fedb873be4d642a1bb56f731a8b87906) |
| TMB2262 | *B. subtilis* 168 *skfEF::mls* | [[4]](#_CTVL0017d8b01ce1e584228933560ec7bdb61c1) |
| TMB6911 | *B. subtilis* DK1042 *epeXEPAB::kan skfA-H::spec* | This study |
| TMB6896 | *B. subtilis* DK1042 *epeXEPAB::kan* | This study |
| TMB1771 | *B. subtilis* 168 *skfABCEFGH::spec* | This study |
| EG494 | *B. subtilis* PY79 *sdpABC-sdpIR::tet* | [[5]](#_CTVL0010d091b8f63ab401ca0dccc32a148c377) |
| TMB3822 | *B. subtilis* 168 *sacA*::P*_liaI_*-*luxABCDE* | [[6]](#_CTVL001ae93c2a78f7e44a3a5ac5142a1ca33fe) |
| TMB4178 | *B. subtilis* 168 *pBS3Clux-PepeX* | [[7]](#_CTVL001b202e2215a67495391a76c48ee890ee7) |
| TMB2841 | *B. subtilis* 168 *sacA*::*luxABCDE (empty vector)* | [[8]](#_CTVL001953cdf72142440e5a9afdc194f4663d9) |
| Nr. 8 | *E. coli* pDG780 (source of *kan* cassette for LFH-PCR) | [[9]](#_CTVL00130fee46ca48c4509b004e2e376fe4124) |
| * resistance designations *mls*: macrolide, lincosamide and streptogramin B; *spec*: spectino-mycin; *kan*: kanamycin *tet*: tetracycline | | |


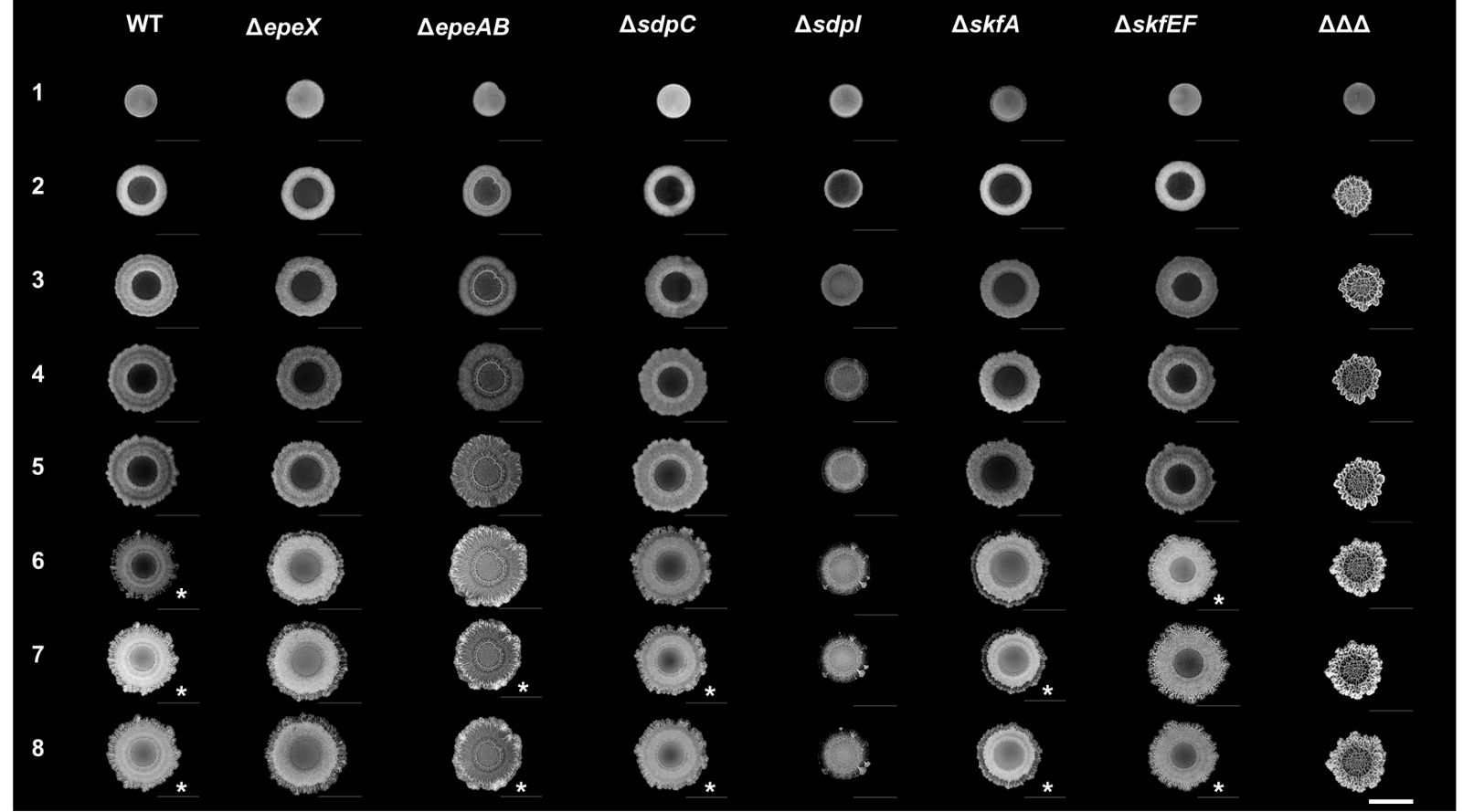


Figure S1 Growth of colonies of B. subtilis WT and cannibalism mutants on MSgg agar plates over 8 days. Genotypes of the strains are denoted in the header, numbers on the left indicate the day of growth. Scale bar indicates 1 cm.


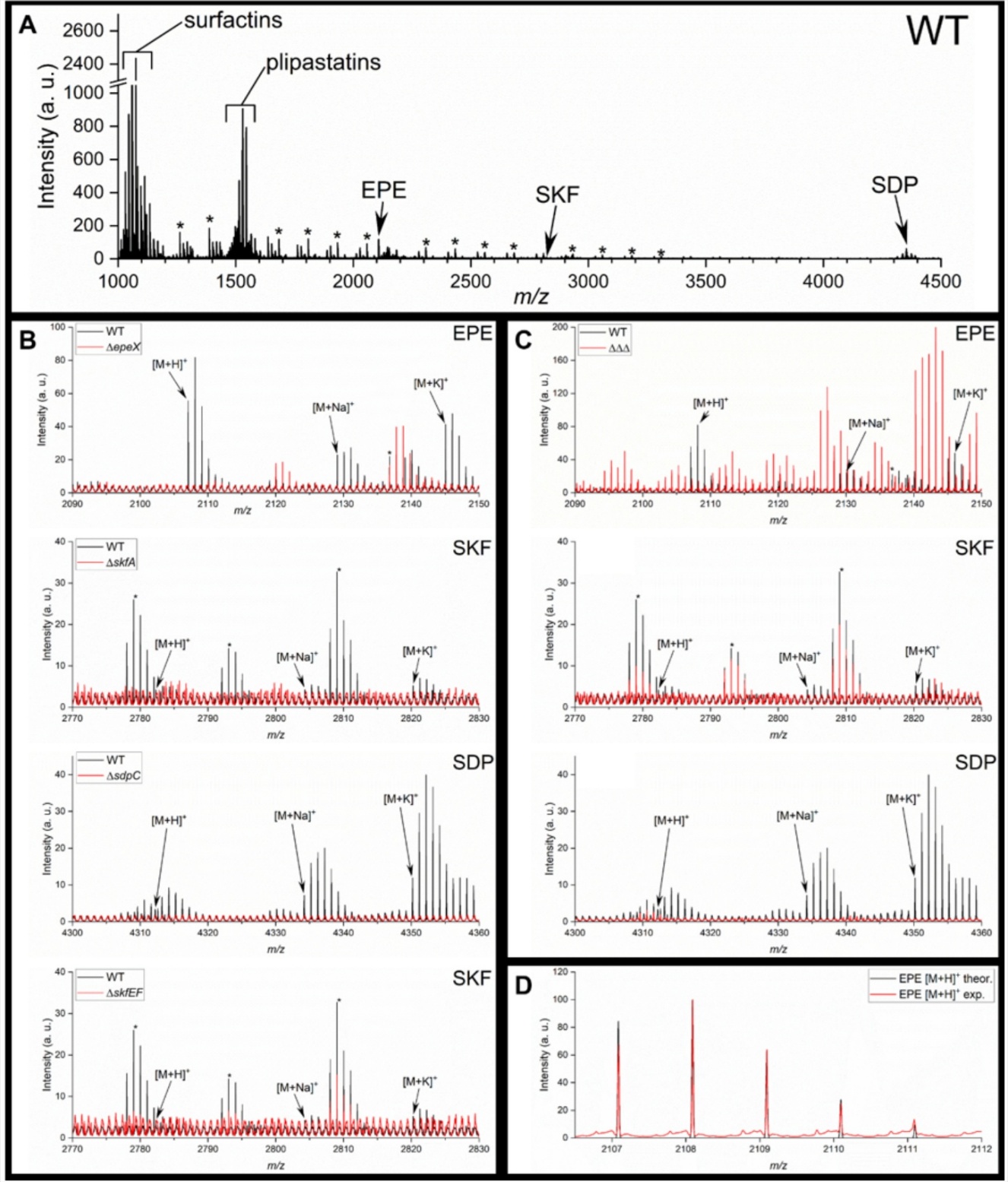


Figure S2 MALDI-MS Imaging mass spectra. [A] Sum mass spectrum acquired from an 8 day old WT B. subtilis biofilm and [B-C] expanded views into m/z regions of interest. WT data are plotted a black traces, mass spectra of selected mutants (indicated at the top left of each panel in B to D) in red. Annotations refer to the [M +H]^+^, [M + Na]^+^ and/or [M + K]^+^ ion species of the toxins as denoted on the top right of the panel. In several cases, the mass spectra corroborate the absence of a particular toxin in a specific knock-out (e.g., the complete loss of all three peptide toxins is found for the hypo-cannibalistic triple knock-out ΔΔΔ in panel C). Please refer to the main text for further details. Tentative assignments are based on accurate mass (~15 ppm agreement with calculated m/z values) and overlap with calculated isotope patterns (illustrated in panel [D] at the example of the [M + H]^+^ species of EPE). As typical for positive ion-mode MALDI-MS measurements, if present, the three peptide toxins were registered as a mixture of [M + H]^+^, [M + Na]^+^, and [M + K]^+^ species, with relative intensities varying according to cation affinities. EPE was for example recorded prominently at for the corresponding ion species calculated m/z values of 2108.09, 2130,07 and 2146.04 presenting the monoisotopic [M + H]^+^, [M + Na]^+^ and [M + K]^+^ ions, respectively; SDP at m/z 4314.22 ([M + H]^+^), 4336.20 ([M + Na]^+^) and 4352.18 ([M + K]^+^), and SKF at m/z 2783.31 ([M + H]^+^), 2805.30 ([M + Na]^+^) and 2821.27 ([M + K]^+^).

Next to the peptide toxins, in some of the mutant strains, many as yet unknown molecules are registered in the MALDI-MSI analysis This is particular notable in the mass spectrum of the hypo-cannibalistic strain of panel C – a finding pointing towards the high potential of the MALDI method as a discovery tool. The overview spectrum of panel A moreover illustrates the simultaneous detection of prominent *B. subtilis* lipopeptides (surfactins and plipastatins with varying hydrocarbon chain lengths). In addition to the endogenous compounds, background ions stemming from the glue used to fix the mixed cellulose ester membranes on the sample plate are eventually also recorded (denoted in A by asterisks).


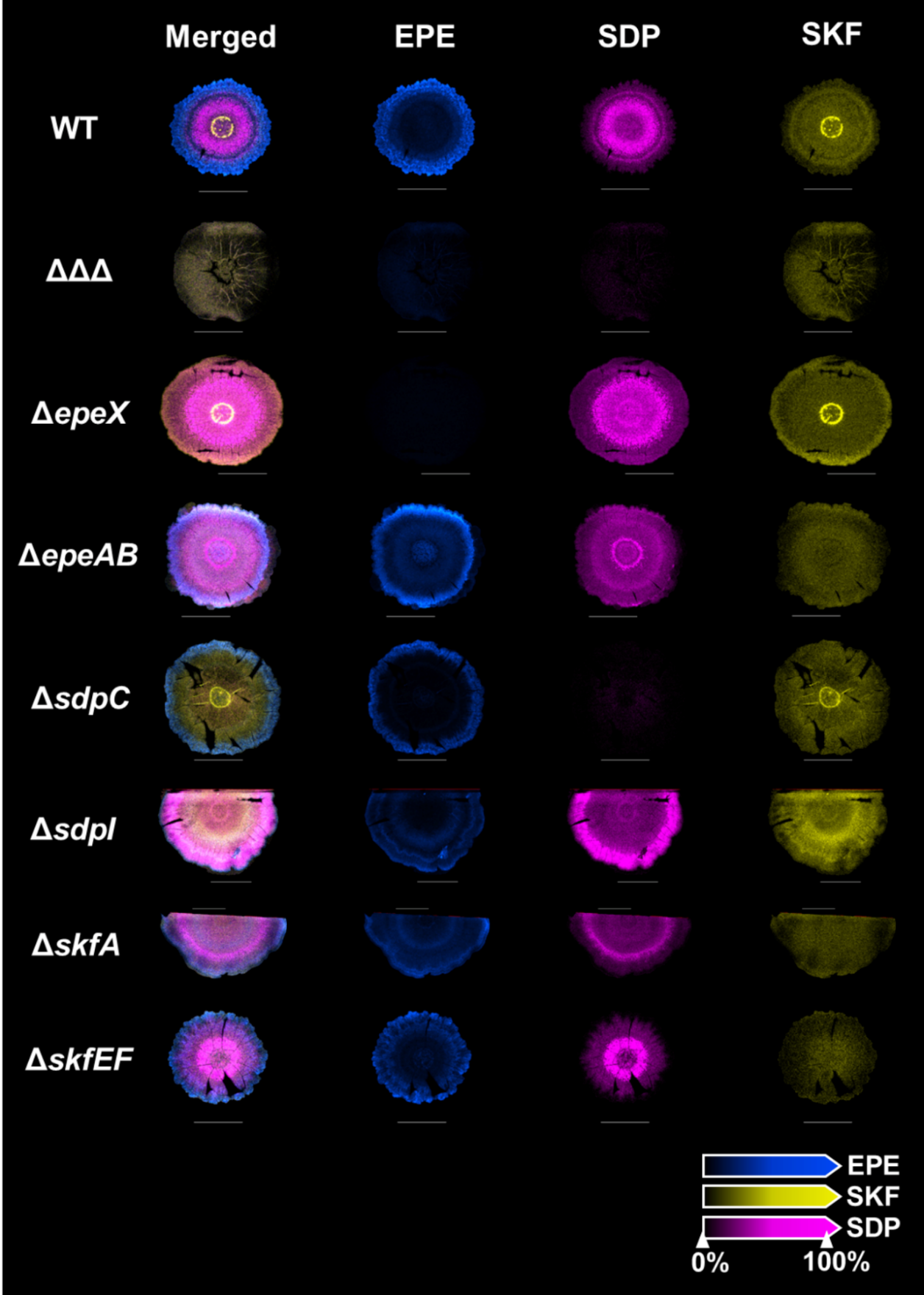
Figure S3 Overview MALDI-MS Imaging results. The false-color images from [M + H]^+^ and [M + K]^+^ signals of all strains used in this study are presented in an overview with merged images and single toxin images provided. Scale bars indicate 1 cm. The recorded *m/z* values are stated in Figure S2.


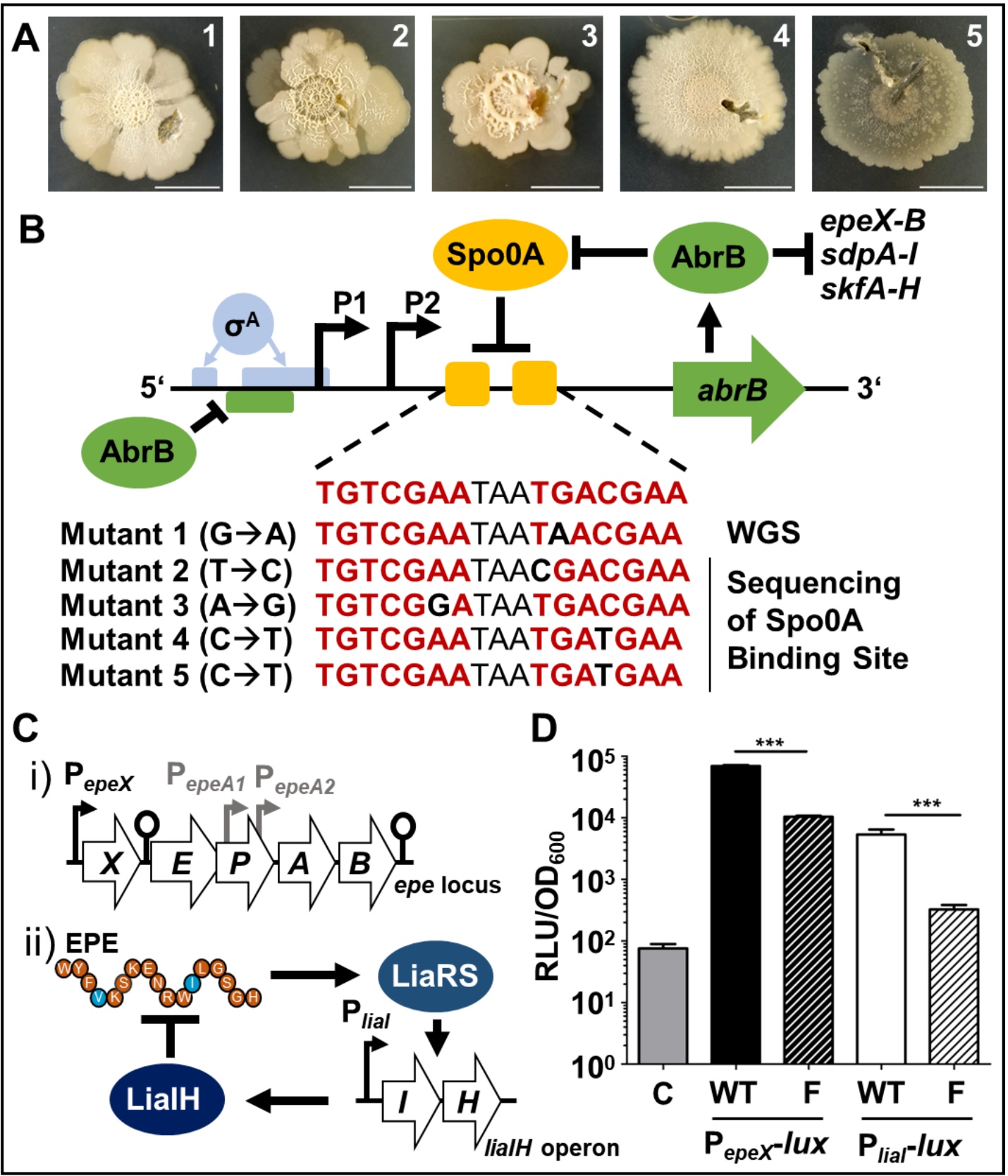


## Figure S4 Analysis of suppressor mutants arising in Δ*epeAB* colony biofilms.

[A] Δ*epeAB* flare mutants 1-5 grown on MSgg agar (day 12 is shown). Scale bars indicate 1 cm. [B] Graphic showing the location of the singularly occurring single nucleotide polymorphisms (SNPs; marked as bold, black letters) in mutants 1-5 within the Spo0A binding sites (red nucleotides) of the *abrB* promoter region and the influence they have on the intricate interplay of Spo0A and AbrB in regard to cannibalistic operon expression. [C] i) Graphic of the *epe* locus and its mapped promoters and transcriptional terminators. ii) Graphic explaining the inducing effect EPE has on P*_liaI_* through recognition by LiaRS and the downstream counteracting mechanism of LiaIH in regard to EPE. [D] Significant differences in activity of P*_epeX_*-lux (black) and P*_liaI_*-lux (white) in the WT and Δ*epeAB* flares (F) showing a reduced expression of *epeX* and the corresponding stress response *liaI* in those suppressor mutants (*** indicate a P value <0.001). C indicates the control (P*_empty_*-*lux*). Strains were measured in biological and technical triplicates. Error bars indicate standard deviation (SD).

## Supplementary Information 1: Testing viability of cells via triplicate analysis and cell sorting of *B. subtilis* wild type (WT)

In microbial flow cytometry there are a number of dyes which are claimed to be useful to measure dead cells. The most often used one is propidium iodide (PI). However, it has been shown that this dye can also label fast growing cells and is therefore not always a reliable marker for dead bacterial cells [[10]](#_CTVL001caacfb9d2ad14c6f9b6f863ae6c5e6c3). Therefore, we performed a more accurate assessment of cell viability by sorting PI-stained cells onto agar plates for re-growth. This re-measurement was performed to demonstrate that PI-stained cells are primarily dead and to show that triplicate measurements support the data of Figure 4 and SI3.

We used the same LB agar plates described in the Materials and Methods section. We used the WT *B. subtilis* DK1042 and measured biological triplicates of whole colonies and biopsies from positions 1 to 6. The respective protocol was as follows: For a liquid preculture, one colony from a preculture plate was transferred to a 100 mL Erlenmeyer flask containing 20 mL of LB medium and was grown at 30°C, 125 rpm for 16 h. After, the liquid preculture was adjusted to an OD600 = 0.1. 5 µL of the OD-adjusted preculture were plated on mixed cellulose ester membranes with 0.22 µm average pore size on MSgg agar, 25 °C, and the cells were grown for 8 d. Two colonies were prepared each for flow cytometric analysis as follows:

• 3x ¼ colony in 1 mL PBS per colony


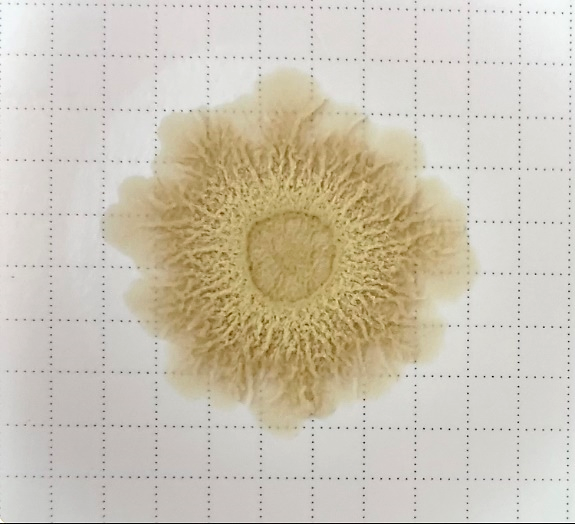

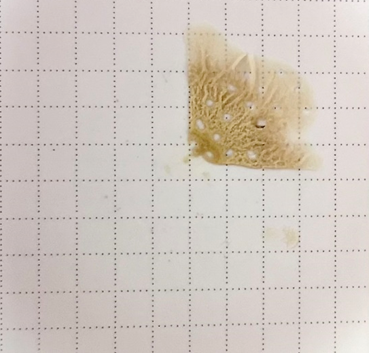
• 3x biopsies at positions 1-6 in 50 µL PBS per colony

**Supplementary Information 1 - Figure 1**: A colony was divided into quarters. 3 quarters were used for triplicates as a whole population. The fourth quarter was 3 times each sampled for triplicate biopsies in positions 1 to 6 (cf. Figure 4A or SI3 Figure 1A for outline). This procedure was performed independently for two colonies.

The samples were treated for flow cytometric analysis and resulting data were evaluated as described in the manuscript (see Materials and Method section).


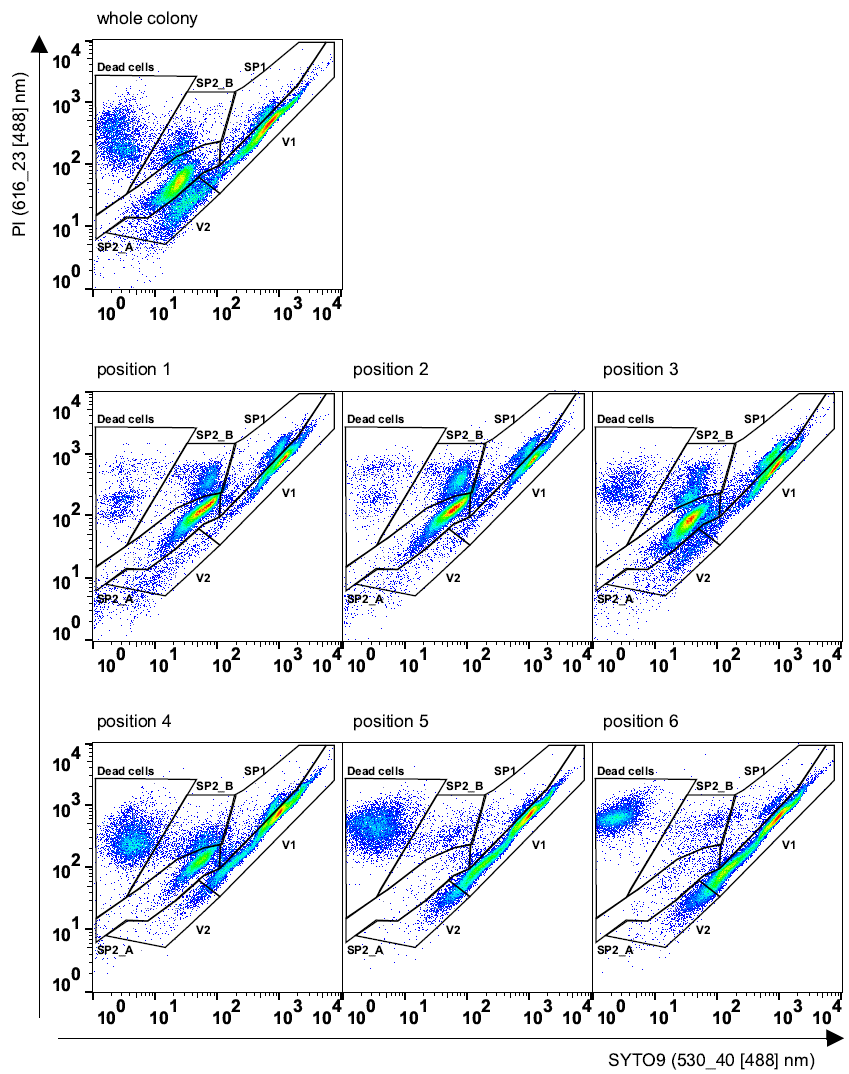


**Supplementary Information 1 - Figure 2**: Flow cytometric measurement of WT *B. subtilis* DK1042 using Syto9/PI. First 2D-plot: measurement of the whole colony. Following 2D-plots: measurement of biopsied positions 1-6. Gates V1 and V2 represent vegetative cells while SP1, SP2A and SP2B represent different vital spore types. 50.000 cells were measured per 2D-plotIn a next step, gates were defined for V1 and V2 as vegetative cell types differing in Syto9 uptake behavior, and SP1, SP2A, and SP2B as different vital spore types, as well as ‘Dead cells’ for PI-stained cells. Cell numbers per gate were counted and compared with findings from Figure 4. The whole colony data for each of the two colonies are shown in Supplementary Information 1 – Table 1. Almost 60% of the events were vital cells, and between 30% and 40% were spores. The number of dead cells was low with about 7% to 9%, the standard deviations (SD) for the respective subpopulations were also low. These findings are similar to the whole community WT data shown in **Supplementary Information 3 – Figure 2,** when the percentages of cells are summed: (67%) vital cells, (28%) spores, and (5%) PI-stained cells. The measurement of triplicate biopsies from 2 independent colonies in positions 1 to 6 revealed very similar data compared to the measurements shown in Figure 4. For colony 1, the vital cells comprised mean 31.3% (position 1), mean 22.5% (position 2), mean 36.1% (position 3), mean 62.2% (position 4), mean 86.2% (position 5), and mean 84.2% (position 6). The spore amounts were mean 65.6% (position 1), mean 75.7% (position 2), mean 59.9% (position 3), mean 25.6% (position 4), mean 3,8% (position 5), and mean 3.5% (position 6). The number of dead cells was between mean 1.5% and mean 12%. Very similar results were obtained by measuring the second colony (**Supplementary Information 1 – Table 1**). These results strongly support those shown in Figure 4. Considering that colonies are highly structured into valleys and mounds, which may contain different types of cells, the variability (SDs) between triplicate biopsies was relatively low.

| **Colony 1**  **Gate** | **WC**  **(%)** | **SD** | **pos. 1 (%)** | **SD** | **pos. 2 (%)** | **SD** | **pos. 3 (%)** | **SD** | **Pos. 4 (%)** | **SD** | **Pos. 5 (%)** | **SD** | **Pos. 6 (%)** | **SD** |
| --- | --- | --- | --- | --- | --- | --- | --- | --- | --- | --- | --- | --- | --- | --- |
| V1 | 46.2 | 2.9 | 29.9 | 1.7 | 21.7 | 0.3 | 33.7 | 3.3 | 59.4 | 9.1 | 76.2 | 4.0 | 67.5 | 9.0 |
| V2 | 9.2 | 1.4 | 1.4 | 0.1 | 0.8 | 0.1 | 2.4 | 0.9 | 2.8 | 2.2 | 10.0 | 6.3 | 16.7 | 8.8 |
| SP1 | 2.0 | 0.1 | 6.9 | 2.0 | 6.2 | 2.4 | 6.8 | 1.3 | 3.7 | 0.2 | 1.5 | 0.2 | 1.2 | 0.6 |
| SP2_A | 31.4 | 1.1 | 52.0 | 3.1 | 61.0 | 4.2 | 48.6 | 4.2 | 18.1 | 7.2 | 0.8 | 0.2 | 1.1 | 0.2 |
| SP2_B | 3.4 | 0.4 | 6.7 | 1.4 | 8.5 | 1.8 | 4.5 | 1.1 | 3.8 | 0.1 | 1.5 | 0.2 | 1.2 | 0.2 |
| Dead cells | 7.2 | 0.5 | 1.9 | 0.1 | 1.5 | 0.1 | 3.7 | 0.8 | 12.0 | 3.5 | 10.0 | 2.9 | 11.9 | 0.9 |
| **Colony 2**  **Gate** | **WC**  **(%)** | **SD** | **pos. 1 (%)** | **SD** | **pos. 2 (%)** | **SD** | **pos. 3 (%)** | **SD** | **Pos. 4 (%)** | **SD** | **Pos. 5 (%)** | **SD** | **Pos. 6 (%)** | **SD** |
| V1 | 47.0 | 2.4 | 27.4 | 1.3 | 21.9 | 1.6 | 25.6 | 0.6 | 35.9 | 2.8 | 61.0 | 11.8 | 60.3 | 13.2 |
| V2 | 11.4 | 0.7 | 3.7 | 1.0 | 2.1 | 0.7 | 1.3 | 0.1 | 3.3 | 1.0 | 15.4 | 4.3 | 27.1 | 13.0 |
| SP1 | 2.3 | 0.1 | 3.9 | 0.4 | 6.3 | 0.4 | 8.5 | 0.8 | 5.6 | 1.0 | 2.8 | 0.9 | 1.5 | 0.3 |
| SP2_A | 26.0 | 0.5 | 54.8 | 3.0 | 58.5 | 1.5 | 52.2 | 0.2 | 45.4 | 2.3 | 6.3 | 2.6 | 1.7 | 0.7 |
| SP2_B | 3.9 | 0.8 | 5.2 | 0.9 | 7.1 | 1.2 | 10.0 | 1.1 | 4.5 | 0.8 | 4.9 | 1.7 | 2.9 | 0.7 |
| Dead cells | 9.0 | 1.5 | 2.6 | 0.6 | 2.5 | 0.5 | 2.1 | 0.4 | 4.8 | 0.9 | 9.1 | 1.8 | 6.0 | 1.8 |

**Supplementary Information 1 – Table 1**: Two independent colonies were each analyzed for proportions of cells from whole communities (WC), and biopsied positions 1-6 (cf. Figure 4 or SI3 Figure 1A for outline). SD: standard deviation from 3 triplicates.

After, cells from positions 5 and 6, which were stained according to the protocol described in [[11]](#_CTVL001d10c91b86fa54356b77c81cb27896f3f) were sorted on 10 x 12 raster = 120 cells on LB agar plates. The LB plates were incubated for regrowth at 30°C for 24 h.


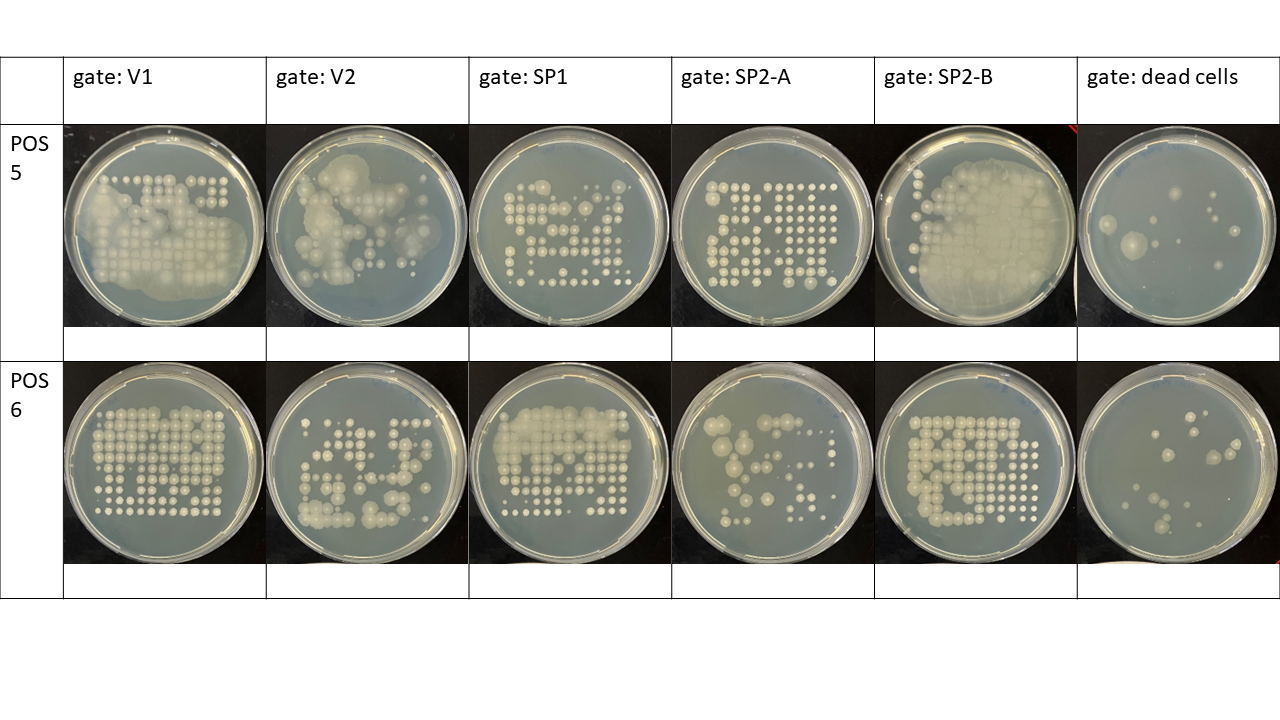
**Supplementary Information 1 - Figure 3**: Cells sorted from subpopulations of biopsied positions 5 and 6 for re-cultivation on agar plates. Upper column: cells gated according to subpopulations V1, V2; SP1; SP2A,B; dead cells. 120 single cells were sorted in an array on the plates. The highest re-cultivation number was reached by gate V1, the lowest recultivation number for the gate of the dead cells (see Supplementary Information 1 – Table 2). Sorting accuracy is 99%.

| nr regrown cells | V1 | V2 | SP1 | SP2_A | SP2_B | dead cells |
| --- | --- | --- | --- | --- | --- | --- |
| Position 5 | 96 | 60 | 76 | 90 | 89 | 12 |
| Position 6 | 111 | 69 | 104 | 45 | 94 | 17 |

**Supplementary Information 1 – Table 2**: Number of cells grown as colonies on plates after sorting. Six subpopulations were sorted per biopsy position.

Regarding the cultivability shown in Supplementary Information 1 – Figure 3 and Table 2: Gate V1 followed by gate SP2-B showed the highest number of re-cultivated cells. Contrarily, only 12 cells and 17 cells for biopsy positions 5 and 6, respectively, out of 120 sorts per plate were found for the PI-stained cells. The relatively high proportion of re-cultivated PI-stained cells may be due to the accuracy of the sorting process (99%) and the ability of PI to enter also live cells when their cell membrane or cell wall begins to rupture. This can occur during rapid growth [[10]](#_CTVL001caacfb9d2ad14c6f9b6f863ae6c5e6c3) or when the membrane is deteriorating but the cells are not yet dead. Nevertheless, it is clear from the data that that the cells in the ‘Dead cell’ gate have a much lower cultivability compared to the vital cells and the spores.

## Supplementary Information 2: Back-gating procedure to verify gate affiliation in double stained 2D-dot plots

When comparing two fluorescence intensities between each other (Syto9 vs. PI), the cells are characterized by the amount of dye they can absorb. For spores this is usually less compared to the vegetative cells because vegetative cells have higher DNA and RNA contents. Therefore, the vegetative cells appear on the right side of the histogram, spores appear in the middle and the dead cells appear on the left because they lack green fluorescence, which is quenched by PI. A way to prove this is the back-gating procedure, where cell types are marked by gates. We used the program FlowJo to do this. The gated cells are back-highlighted in a Syto9 vs. FSC visualization, which can be done with each subpopulation. Back-gating shows the spores on the right side of the histogram again because they provide a higher scatter signal due to their intrinsic spore density.


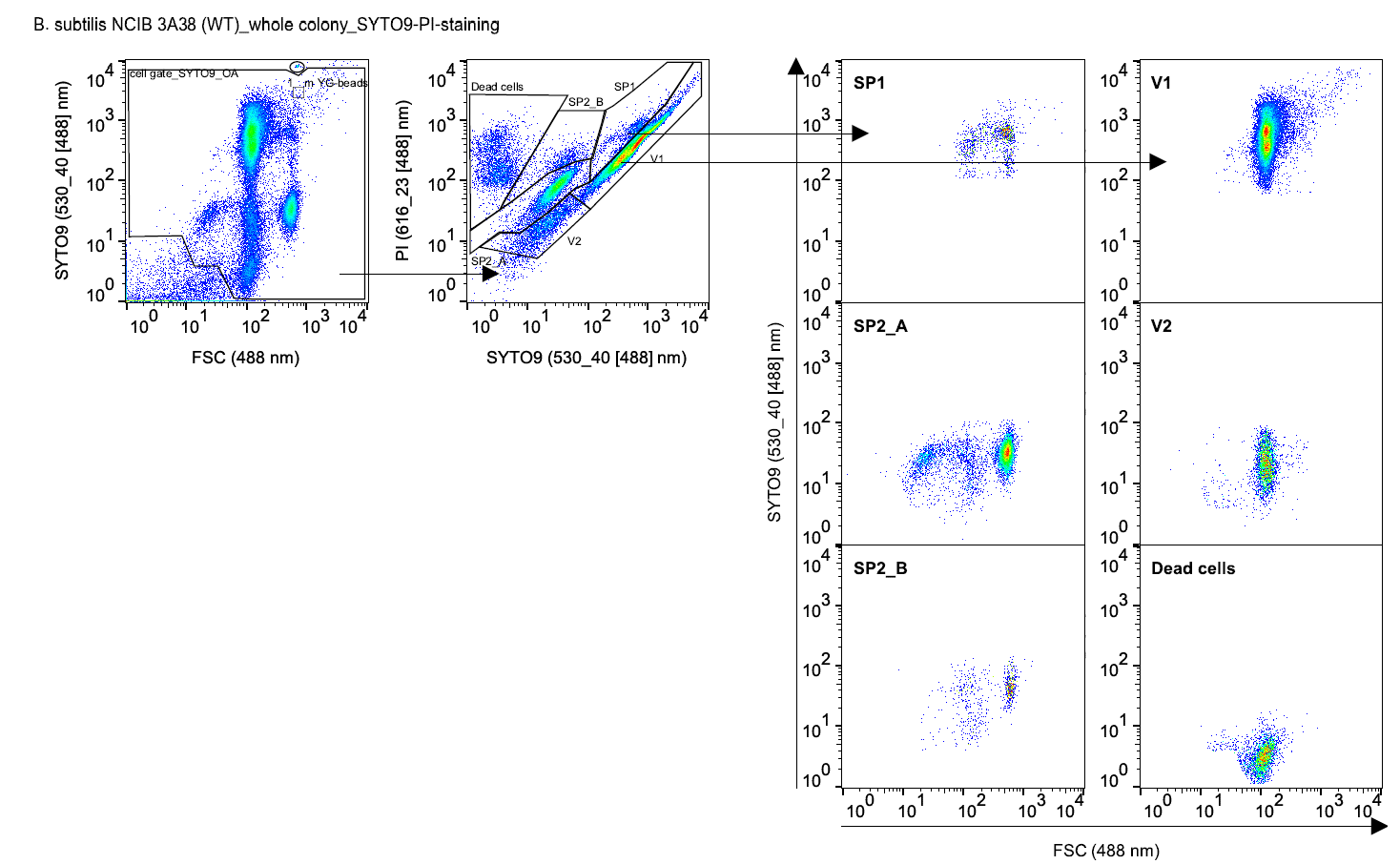


**Supplementary Information 2 - Figure 1**: Flow cytometric measurement of whole colony WT *B. subtilis* DK1042 using Syto9 vs. PI. Left: visualization of the whole colony as Syto9 vs. FSC 2D-dot plot. Middle: visualization of the whole colony as Syto9 vs. PI 2D-dot plot. Right: back-gating of each of the six gates to generate new Syto9 vs. FSC 2D-dot plots. Gates V1 and V2 represent vegetative cells while SP1, SP2A and SP2B represent different vital spore types. 50.000 cells were measured per 2D-dot plot.

The back-gating enables detection of the position of the cells within the respective gates on the Syto9 vs. FSC 2D-dot plot. For example., the dead cells show nearly no Syto9 fluorescence while having the same size as the vegetative cells, and thus have lower scatter compared to spores. Back-gating allows the spores to be clearly differentiated from the vegetative cells. Back-gating can also be used to sort specific cell gates.

##
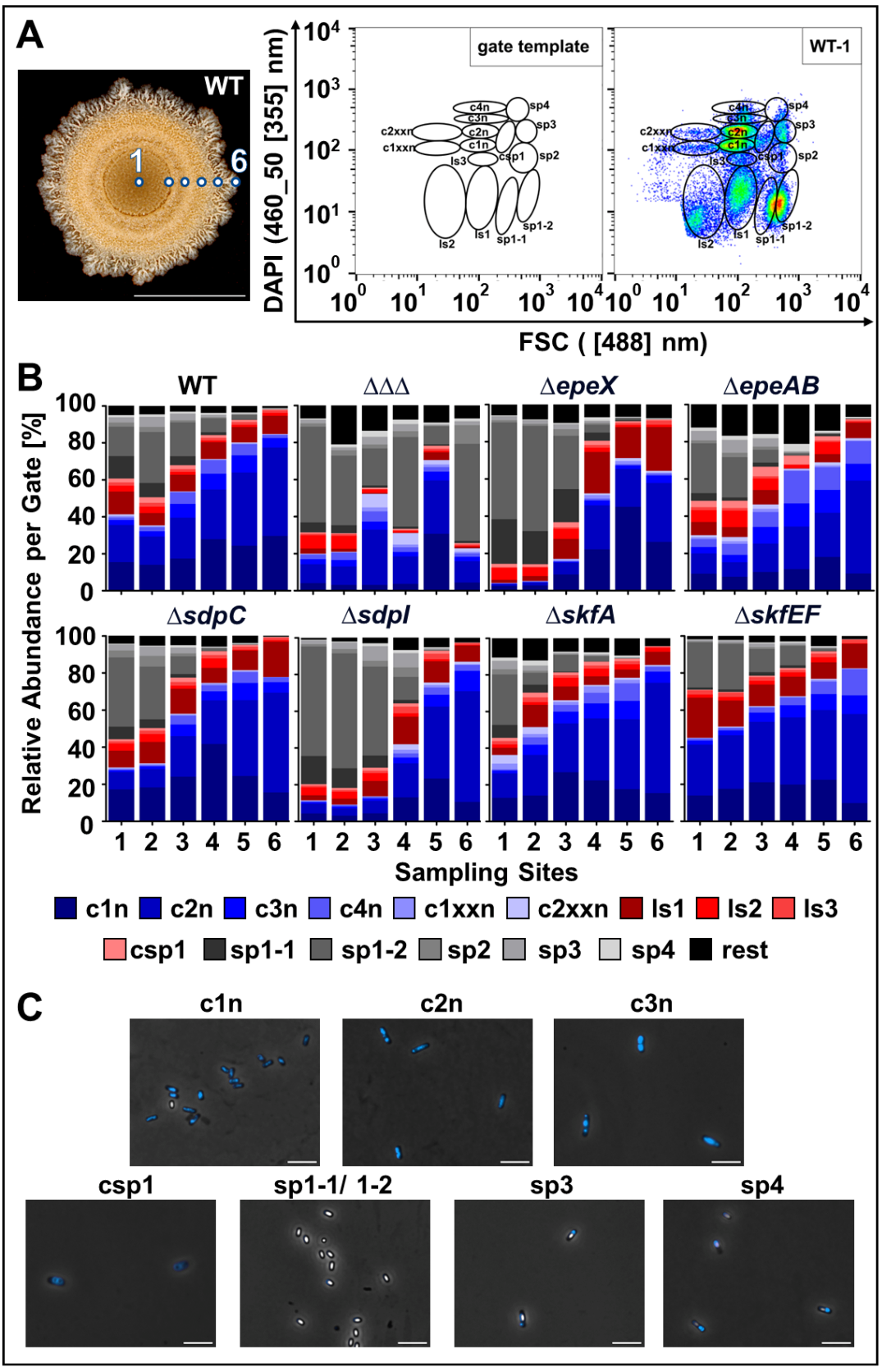
Supplementary Information 3: Colony biopsy in combination with microbial flow cytometry and cytometric fingerprinting

Supplementary Information 3 - Figure 1: Distribution of cell types across B. subtilis colonies regarding cannibalism after DAPI staining. [A] Sampling sites (1-6) across an exemplary WT colony are shown on the left. Scale bar indicates 1 cm. On the right, gate templates for analysis and sorting are depicted, as well as an exemplary dataset of flow cytometric measurement of cells present in sampling site 1 of the WT. DAPI signal is plotted against forward scatter (FSC). [B] Overview of flow cytometric analysis results are summarized in bar graphs for each strain, where the relative abundance of cells per gate is plotted against sampling sites. c1n – c2xxn indicate vegetative cells, and ls1 – ls3 indicate less stained cells, meaning that they do not take up DAPI easily. Csp1, sp1-1 – sp4 represent different spore types. [C] Microscopy images of sorted cells from the WT representing cytometrically analyzed cell types from respective gates. Images captured using phase contrast microscopy are overlaid with those captured using fluorescence microscopy. Scale bars indicate 5 µm. The cells were treated and sorted using a procedure published in Nature Protocols [[12]](#_CTVL0019cf50824414d4963a43ad1cebec69da0).


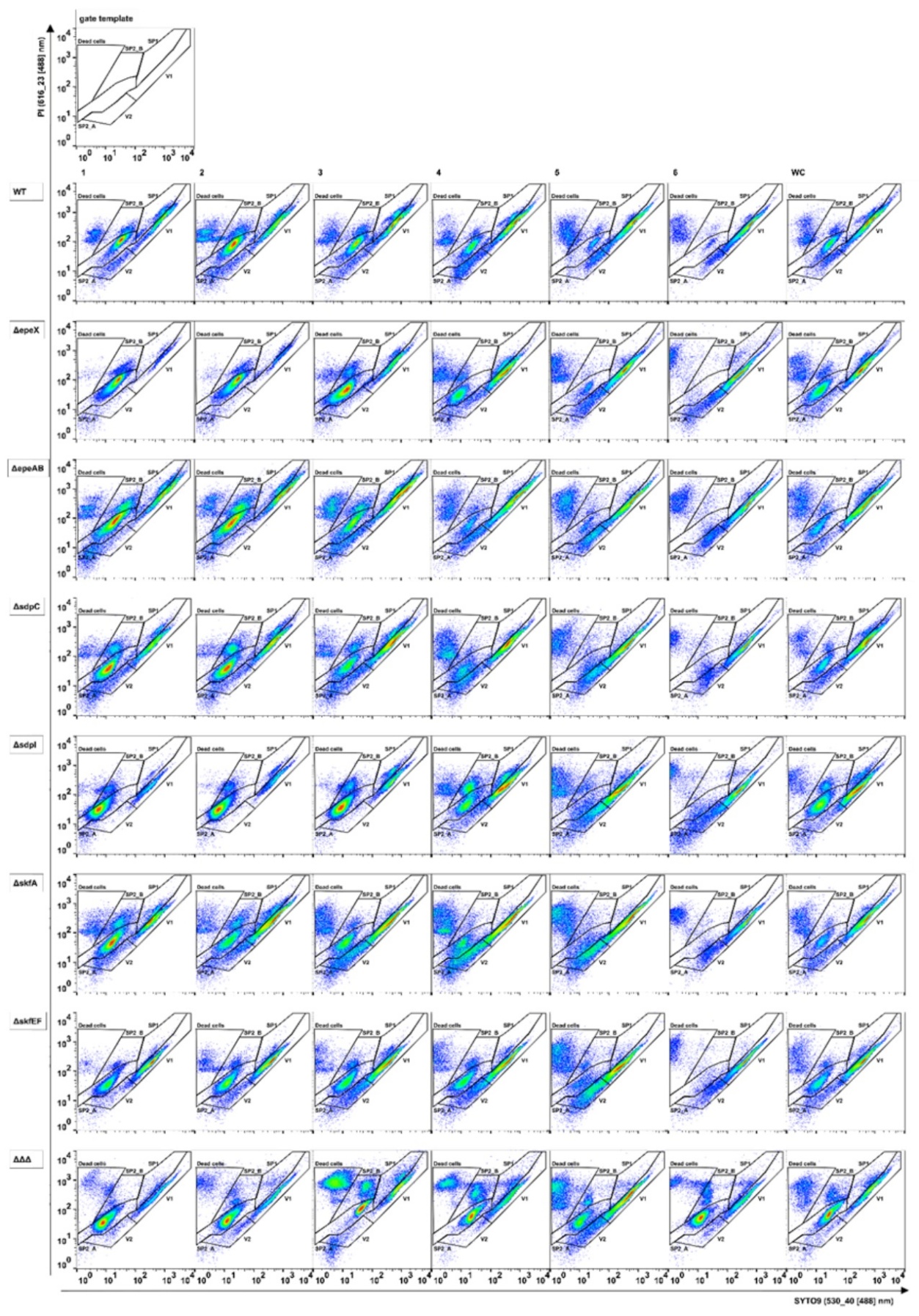
Supplementary Information 3 – Figure 2: Overview of flow cytometric results for SYTO9/PI staining. Top left shows the gate template. Datasets for each strain are shown in a row, starting with sampling site 1-6, followed by the data obtained for the whole colony (WC). 2D plots depict SYTO9 vs. PI data [rel. FI].


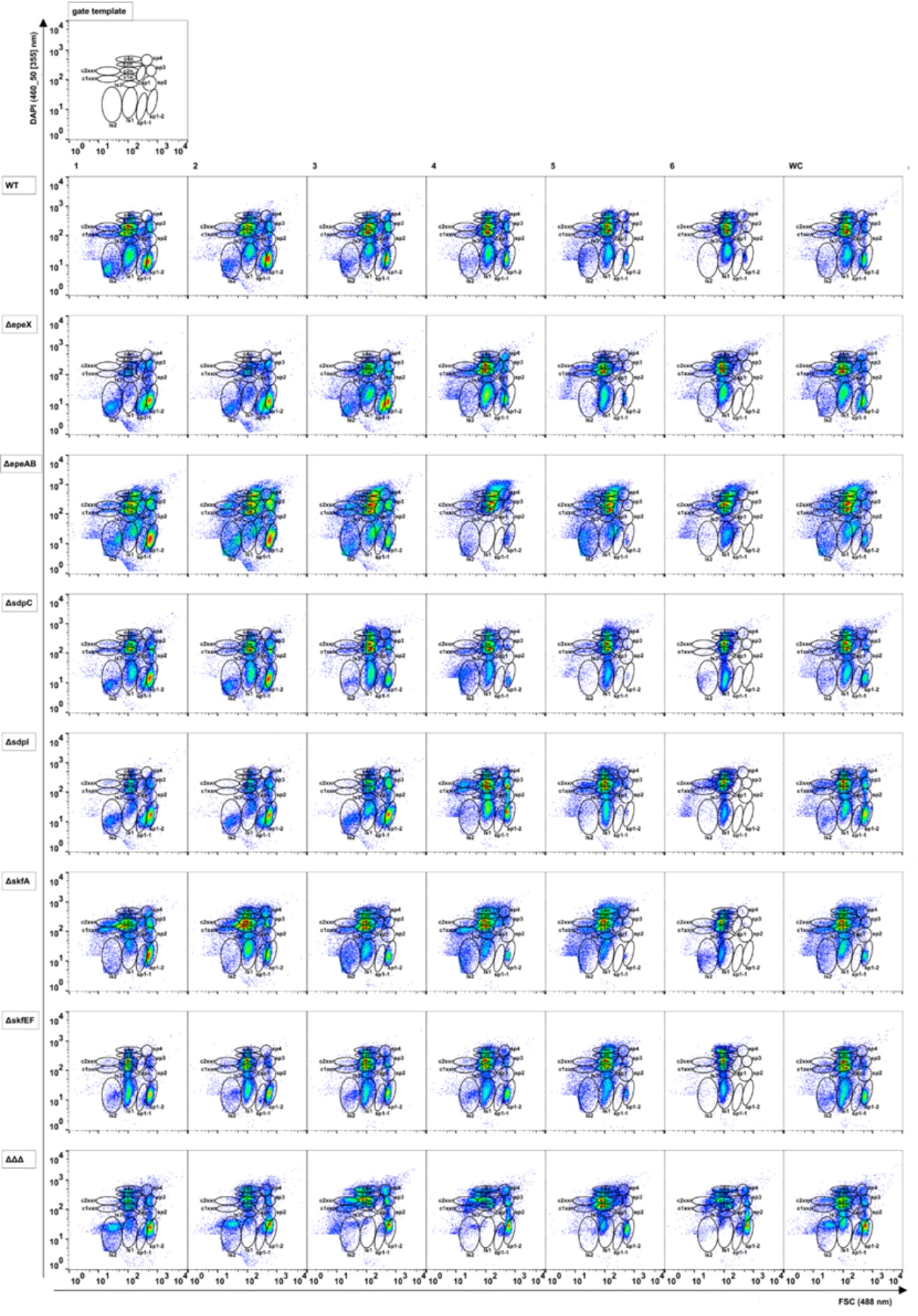
Supplementary Information 3 – Figure 3: Overview of flow cytometric results for DAPI staining. Top left shows the gate template. Datasets for each strain are shown in a row, starting with sampling site 1-6, followed by the data obtained for the whole colony (WC). 2D plots depict FSC vs. DAPI data [rel. FI].

## Supplementary Information 4: Staining procedures for flow cytometry

1. **Live/dead cell staining for flow cytometry**

Single-cell staining was performed in an identical manner to that described in reference [[11]](#_CTVL001d10c91b86fa54356b77c81cb27896f3f). The method was exactly performed as described previously: To distinguish between live and dead cells, a combination of SYTO9 (all cell green-fluorescent membrane permeant nucleic-acid stain; Ref. S-34854, *Thermo Fisher Scientific*, Waltham, MA, USA) and propidium iodide (PI, indicator for compromised cell membranes and cell walls; Ref. P4170, *Merck KGaA*—Sigma Aldrich, Darmstadt, Germany) was applied to live cells. A working solution of 10 µM SYTO9 (in 0.85% NaCl; 0.2 µm filtered) was freshly prepared each day from a 5 mM stock solution and stored on ice. To achieve a final concentration of 0.5 µM SYTO9 per sample, 7.9 µl of the SYTO9 working solution was added to 150 mL of adjusted cell suspension. In addition, PI (105 µM stock solution in 0.85% NaCl) was added at a concentration of 1.8 µM per sample. During staining, samples were kept for 12 min at RT. Following this, 3 µl reference beads were added for biopsy sites 1–4 and 1 µl reference beads for biopsy site 5 to keep the cell-bead ratio (1 µm yellow-green (YG) fluorescent Fluospheres; Ref. F-8823, *Thermo Fisher Scientific*, Waltham, MA, USA) as an internal standard. The entire procedure was completed within 15 min.

1. **DNA cell staining for flow cytometry**

Single-cell staining was performed in an identical manner to that described by Abbaszade *et al*. [[11]](#_CTVL001d10c91b86fa54356b77c81cb27896f3f). The method was exactly performed as described: DAPI staining required cell fixation The NaCl/NaN_3_/EtOH fixation was carried out by adding 237.5 µL of 30% NaCl, 17.5 µL of 20% NaN_3_ (both *Merck KGaA*, Darmstadt, Germany), and 50 µL of 70% EtOH (*Chemsolute*, Renningen, Germany) to 50 µL of the bacterial sample. This resulted in final concentrations of 20% NaCl, 1% NaN_3_, and 10% EtOH, with an incubation time of 10 min. Following fixation, a centrifugation step (5 min at 5,000 x g) was done, supernatant was removed by pipetting and 50µL PBS was added to resolve the pellet of the fixed cells. Subsequently, 100 µL of 5 µM DAPI staining solution were added to 50 µL of the cell suspension, incubated for 10 min and measured immediately.

References

[1] Konkol M.A., Blair K.M., Kearns D.B., 2013. Plasmid-Encoded ComI Inhibits Competence in the Ancestral 3610 Strain of *Bacillus subtilis*. Journal of Bacteriology 195(18):4085–93. https://doi.org/10.1128/JB.00696-13.

[2] Koo B.-M., Kritikos G., Farelli J.D., Todor H., Tong K., Kimsey H. et al., 2017. Construction and Analysis of Two Genome-Scale Deletion Libraries for *Bacillus subtilis*. Cell Syst 4(3):291-305.e7. https://doi.org/10.1016/j.cels.2016.12.013.

[3] Butcher B.G., Lin Y.-P., Helmann J.D., 2007. The *yydFGHIJ* Operon of *Bacillus subtilis* Encodes a Peptide that Induces the LiaRS Two-Component System. Journal of Bacteriology 189(23):8616–25. https://doi.org/10.1128/JB.01181-07.

[4] Höfler C., Heckmann J., Fritsch A., Popp P., Gebhard S., Fritz G. et al., 2016. Cannibalism Stress Response in *Bacillus subtilis*. Microbiology (United Kingdom) 162(1):164–76. https://doi.org/10.1099/mic.0.000176.

[5] Ellermeier C.D., Hobbs E.C., Gonzalez-Pastor J.E., Losick R., 2006. A Three-Protein Signaling Pathway Governing Immunity to a Bacterial Cannibalism Toxin. Cell 124(3):549–59. https://doi.org/10.1016/j.cell.2005.11.041.

[6] Popp P.F., Dotzler M., Radeck J., Bartels J., Mascher T., 2017. The Bacillus BioBrick Box 2.0: Expanding the Genetic Toolbox for the Standardized Work with *Bacillus subtilis*. Sci Rep 7(1):15058. https://doi.org/10.1038/s41598-017-15107-z.

[7] Popp P.F., Friebel L., Benjdia A., Guillot A., Berteau O., Mascher T., 2021. The Epipeptide Biosynthesis Locus *epeXEPAB* Is Widely Distributed in *Firmicutes* and Triggers Intrinsic Cell Envelope Stress. Microbial Physiology 31(3):306–18. https://doi.org/10.1159/000516750.

[8] Pinto D., Vecchione S., Wu H., Mauri M., Mascher T., Fritz G., 2018. Engineering Orthogonal Synthetic Timer Circuits Based on Extracytoplasmic Function σ Factors. Nucleic Acids Res 46(14):7450–64. https://doi.org/10.1093/nar/gky614.

[9] Guérout-Fleury A.M., Shazand K., Frandsen N., Stragier P., 1995. Antibiotic-Resistance Cassettes for *Bacillus subtilis*. Gene 167(1-2):335–6. https://doi.org/10.1016/0378-1119(95)00652-4.

[10] Shi L., Günther S., Hübschmann T., Wick L.Y., Harms H., Müller S., 2007. Limits of Propidium Iodide as a Cell Viability Indicator for Environmental Bacteria. Cytometry A 71(8):592–8. https://doi.org/10.1002/cyto.a.20402.

[11] Abbaszade G., Stückrath K., Müller S., 2025. Bacterial Colony Biopsies: Spatial Discrimination of Heterogeneous Cell Types by Cytometric Fingerprinting. Methods Ecol Evol. https://doi.org/10.1111/2041-210X.70022.

[12] Cichocki N., Hübschmann T., Schattenberg F., Kerckhof F.-M., Overmann J., Müller S., 2020. Bacterial Mock Communities as Standards for Reproducible Cytometric Microbiome Analysis. Nature Protocols 15(9):2788–812. https://doi.org/10.1038/s41596-020-0362-0.
